# The solitary nucleus connectivity to key autonomic regions in humans

**DOI:** 10.1111/ejn.15691

**Published:** 2022-06-21

**Authors:** Julia Forstenpointner, Anne Margarette S. Maallo, Igor Elman, Scott Holmes, Roy Freeman, Ralf Baron, David Borsook

**Affiliations:** 1 Center for Pain and the Brain, Boston Children’s Hospital, Department of Anesthesia, Critical Care and Pain Medicine, Harvard Medical School, Boston, Massachusetts; 2 Division of Neurological Pain Research and Therapy, Department of Neurology, University Hospital Schleswig-Holstein, Kiel, Germany; 3 Cambridge Health Alliance, Harvard Medical School, Cambridge, Massachusetts, USA; 4 Department of Neurology, Beth Israel Deaconess Medical Center, Harvard Medical School, Boston, Massachusetts, USA; 5 Department of Radiology and Psychiatry, Massachusetts General Hospital, Harvard Medical School, Boston, Massachusetts, USA

**Keywords:** connectome, interoceptive, laterality, magnetic resonance imaging, medulla oblongata, viscero-sensory

## Abstract

The nucleus tractus solitarius (NTS) is a key brainstem structure relaying interoceptive peripheral information to the interrelated brain centres for eliciting rapid autonomic responses and for shaping longer-term neuroendocrine and motor patterns. Structural and functional NTS’ connectivity has been extensively investigated in laboratory animals. But there is limited information about NTS’ connectome in humans. Using MRI, we examined diffusion and resting state data from 20 healthy participants in the Human Connectome Project. The regions within the brainstem (*n* = 8), subcortical (*n* = 6), cerebellar (*n* = 2) and cortical (*n* = 5) parts of the brain were selected via a systematic review of the literature and their white matter NTS connections were evaluated via probabilistic tractography along with functional and directional (i.e. Granger causality) analyses. The underlying study confirms previous results from animal models and provides novel aspects on NTS integration in humans. Two key findings can be summarized: (1) the NTS predominantly processes afferent input and (2) a lateralization towards a predominantly left-sided NTS processing. Our results lay the foundations for future investigations into the NTS’ tripartite role composed of interoreceptors’ input integration, the resultant neurochemical outflow and cognitive/affective processing. The implications of these data add to the understanding of NTS’ role in specific aspects of autonomic functions.

## 1 | INTRODUCTION

The nucleus of the solitary tract (NTS) is a key integrator for a variety of autonomic functions that comprises a group of sensory nuclei, located in the dorsal medulla oblongata. It serves as the first central relay of the interoceptive peripheral input from pulmonary, gastrointestinal, laryngeal, tracheal, cardiac as well as baro- and chemo-receptors’ afferents (Altschuler et al., 1989; Chan et al., 2000; Ciriello, 1983; Ciriello et al., 1981; Housley et al., 1987; Jordan & Spyer, 1986; Kalia et al., 1984; Kalia & Mesulam, 1980; Wallach & Loewy, 1980) to brainstem areas, for example, the largest noradrenergic nucleus, the locus coeruleus (Lopes et al., 2016) and higher corticolimbic structures, for example, the insula and hypothalamus (Travagli, 2007). These inputs follow a viscerotopic organization (Jordan & Spyer, 1986; Kalia et al., 1984) to elicit reflexive autonomic responses within the brainstem via projections from the NTS that are additionally modulated by input from other CNS sites, shaping long-term neuroendocrine and motor patterns (Travagli et al., 2006).

Notably, the lateralization (i.e. towards right or left) of autonomic functions of the CNS has been investigated previously (Guo et al., 2016; Thayer & Lane, 2009). For instance, in the CNS, some studies suggest lateralization in the insula (Lacuey et al., 2018; S. Oppenheimer & Cechetto, 2016; S. M. Oppenheimer et al., 1992; Ruiz Vargas et al., 2016)—within the right for sympathetic control and the left for parasympathetic control; however, this has not been confirmed by others (Chouchou et al., 2017, 2019; Marins et al., 2017; Szurhaj et al., 2015). The right prefrontal cortex is also reportedly associated with inhibitory cardiac function mediated via inputs from the vagal nerve (Ahern et al., 2001; Aron et al., 2004, 2014). Additionally, primarily right-sided cortical tracts innervate the myocardium (Ter Horst & Postema, 1997), which has been corroborated by the retrograde labelling of nucleus ambiguus neurons (right 75% > left 25%) via injection of horseradish peroxidase into the sinoatrial node (Chuang et al., 2004). The above examples indicate that dominance of autonomic function may have a lateralized component, but little is known whether afferent or efferent connections from or towards the NTS follow tracts that are physiologically lateralized in humans. A uniform explanation of these observations pertains to the fact that lateralization may either follow the demand of the effector organ or results from a lateralized receptor density. Given that the NTS is one of the main afferent relay stations for autonomic sensory inputs (Travagli, 2007), it is vital to understand how the connections of the NTS and their potential lateralization may impact the CNS in the event of autonomic dysfunction.

Furthermore, the NTS is among the first regions to receive sensory input from peripheral organs (Jean, 1991; McDougall et al., 2009), which underlines its vulnerability towards sensitization and functional alterations in the event of autonomic dysfunction. There is evidence that stimulation of secondary neurons in the NTS alter baroreceptor function and thus central nervous system (CNS) processing (Seller & Illert, 1969). Additionally, plasticity changes within the NTS have been suggested in the context of lung and airway reflexes (Bonham et al., 2006), after episodic ozone exposure of primates (C. Y. Chen et al., 2003), as well as in sudden death of mice with acquired temporal lobe epilepsy (Derera et al., 2017).

Together, the above examples indicate that autonomic function may have a lateralized component, but little is known whether afferent (i.e. from the NTS to upstream CNS regions) or efferent NTS connections follow tracts that are physiologically lateralized in humans. In particular, the role of the NTS as a sensory integrator may be prone to plasticity changes, which may alter lateralization patterns and in turn affect regions and structures connected to the NTS. The latter notion, however, cannot be addressed without establishing a physiological connectivity map of the NTS. Thus, an understanding of not just NTS connectivity but also the lateralization of afferent and efferent connections may add to our understanding of clinical observations in autonomic disease as well as treatment effects of (e.g. vagus nerve stimulation) (M. Chen et al., 2015).

Although animal studies discussed above have advanced our understanding of central processing in the NTS of both afferent input (McDougall et al., 2017; Ricardo & Koh, 1978; Shin et al., 2008; Viltart et al., 2006) and efferent output (Blessing et al., 1981; Ciriello & Caverson, 1986; Kapp et al., 1985; Loewy et al., 1981; Ross et al., 1981; Shipley, 1982), we still lack a clear understanding of afferent versus efferent processing in the NTS in humans. The majority of the above-mentioned morphological and physiological findings are based on antero-/retrograde tracing or the measurement of autonomic parameters (heart rate, blood pressure, respiratory rhythm etc.) in mammals (e.g. rodents, cats and monkeys), but not in humans. In recent years, however, non-invasive neuroimaging has made it possible to study the NTS in both animals and humans (see Table S1). Such studies have highlighted the complex nature of NTS functionality, showing activity differences in the NTS involving various organ divisions. However, there are major challenges in translating findings from animal studies into human research as outlined in the discussion. To date, a systematic analysis on how the human NTS is structurally and functionally connected to neighbouring structures within the brainstem or distant subcortical/cortical regions is missing.

In this paper, the connections to and from the NTS in the human brain were studied using structural and functional connectivity analyses in a group of 20 healthy subjects. In order to identify and study key autonomic regions connected to the NTS, considerations were based on the following approaches: First, we conducted a systematic review of the literature in humans and animals to identify connections from the NTS towards other central and peripheral autonomic regions; second, based on the literature, we evaluated possible direct white matter connections from the human NTS towards key autonomic regions within the brainstem, subcortical and cortical regions via high-angular resolution diffusion imaging (HARDI). Third, we assessed the functional NTS connections and their likely directionality (i.e. input/output) via functional connectivity and Granger causality modelling of resting state fMRI. Lastly, we discuss the potential lateralized connectivity of the NTS to various autonomic systems.

## 2 | METHODS

### 2.1 | Systematic literature review

A systematic literature research to identify MRI-based research of the NTS was conducted via PubMed (https://pubmed.ncbi.nlm.nih.gov/) on the 15 March 2021. The following search keywords were used: *([NTS] OR [nucleus tractus solitary] OR [nucleus tractus solitarii] OR [solitary nucleus]) AND ([MRI] OR [DTI] OR [magnet resonance imaging] OR [diffusion tensor imaging])*. The selection of suitable manuscripts obeyed the following exclusion criteria: (1) exclusion of reviews and case reports and (2) exclusion of manuscripts that were falsely identified, that is, mostly due to the diverse use of NTS as an abbreviation.

All studies identified as suitable in the literature research were displayed in Table S1. Regions of interest (ROIs) identified from this systematic review were used in the imaging analyses described below.

### 2.2 | Magnetic resonance imaging (MRI)

#### 2.2.1 | Participants

In total, 20 unrelated subjects (age range: 22–35; 15 females) were randomly chosen from the Human Connectome Project (HCP). In addition, to obtaining imaging data from the HCP database, demographic data (sex, range of age in years) were collected (see Table S2). The protocol of the HCP was implemented according to the Declaration of Helsinki (Association, 2013) and approved by the Institutional Review Board of Washington University in St. Louis (MO, USA).

#### 2.2.2 | MRI data acquisition

All acquisition scans followed HCP protocols, which can be found in more detail in the reference manual of the WU-Minn HCP 1200 Subjects Data Release (https://db.humanconnectome.org). Briefly, the images used here were acquired on a 3T Siemens Skyra MRI Scanner (Erlangen, Germany) with a 32-channel head coil (Glasser et al., 2016; Van Essen et al., 2012). The HCP Skyra uses a standard set of Siemen’s shim coils (up to 2nd order). However, in comparison with the standard version, the customized HCP Skyra increases the maximum gradient strength from 40 to 100 mT/m due to the combination of a gradient coil and gradient power amplifiers. This set up specifically benefits diffusion imaging and provides significant gains over the standard 40 mT/m on theoretical grounds (Uğurbil et al., 2013). For each subject, multi-shell HARDI and resting-state data for tractography and functional connectivity were analysed, respectively. The anatomical images were acquired using the MPRAGE sequence (total acquisition time = 460 s, 0.7 mm^3^ isotropic resolution; TR/TE = 2400/2.14 ms, FOV = 224 × 224 mm). The multi-shell HARDI images were acquired using a single-shot 2D spin-echo EPI sequence (multiband factor = 3, total acquisition time = 59 min with six runs approximately 9 min 50 s per run, 1.25 mm^3^ isotropic resolution, TR/TE = 5520/89.5 ms, FOV = 210 × 180 mm and b = 1000, 2000 and 3000 s/mm^2^, each with 90 directions and each with L-R phase-encode). A complete set of opposite phase-encode directions for each gradient encoding was also acquired. Additionally, six b = 0 images were interspersed in each run. The resting-state data were acquired with a gradient-echo EPI sequence (multi-band factor = 8, acquisition time of one run 14 min 33 s corresponding to 1200 volumes, 2.0 mm^3^ isotropic resolution; TR/TE = 720/33.1 ms, FOV = 208 × 180 mm).

#### 2.2.3 | Preprocessing of MRI data

All preprocessing of MRI data was done through the Boston Children’s Hospital’s High-Performance Computing Resources Cluster Enkefalos 2. Software used in the project was installed and configured by BioGrids (Morin et al., 2013).

##### HARDI

All HARDI images were preprocessed using Mrtrix v3.0.1, 64 bit release version, built 1 July 2020, using Eigen 3.3.7 (J. D. Tournier et al., 2019) with the following steps: removal of noise (Cordero-Grande et al., 2019; Veraart, Fieremans, et al., 2016; Veraart, Novikov, et al., 2016), Gibbs ringing artefact (Kellner et al., 2016), eddy (Skare & Bammer, 2009) and susceptibility-induced distortions (Andersson et al., 2003; Andersson & Sotiropoulos, 2016; Skare & Bammer, 2009; Smith et al., 2004). The eddy and susceptibility distortion corrections were performed using the Mrtrix wrapper script, dwifslpreproc, that uses eddy, topup and apply topup. Next, the response function was estimated using the Dhollander algorithm (Dhollander et al., 2019) followed by estimation of the fibre orientation distributions (FOD) using the multi-shell, multi-tissue constrained spherical deconvolution (Jeurissen et al., 2014). The FOD was then used in probabilistic tractography (Tournier et al., 2010). Given the differences across ROI size, seed density was kept constant at 100 seeds per voxel to control the number of tracking trials per ROI.

##### Resting-state

All functional images were processed in each subject’s native volumetric space. fMRI BOLD data were preprocessed, using AFNI ‘Claudius’ v 19.2.01 (Cox, 1996). Pre-processing for the functional connectivity analysis included the following steps: all volume images were registered to the volume image with the least motion and outlier voxels, and the volume-registered 4D series data were co-registered to the participant’s b = 0 image from HARDI. The time series of each voxel was despiked, corrected for slice-time acquisition offset, scaled to a mean of 100 a.u. and maximum of 200 a.u., band-passed for frequencies 0.01–0.1 Hz, and smoothed with a Gaussian kernel (FWHM = 4 mm). The mean motion in six directions and its derivative, as well as the mean signal from the white matter and cerebrospinal fluid voxels were regressed out of each voxel’s time series. Finally, time points with motion greater than 0.4 mm or with greater than 10% of outlier voxels were censored to zero. The mean time course of all voxels within a region of interest (ROI) mask was extracted and used in further analysis (see below for list of ROI). The same resting-state data were preprocessed for Granger causality analysis to determine the plausible directionality of the functional connection between the bilateral NTS and each ROI. The same steps above were applied without despiking, scaling, censoring and bandpass filtering for pre-processing consistent with suggestions for Granger causality analysis of fMRI BOLD signal (Seth, 2010).

#### 2.2.4 | Localization, parcellation and segmentation of regions

##### Subcortical and cortical regions

The localization of subcortical and cortical regions followed an automated approach. The Freesurfer parcellation of the cortex was used to create masks for each individual in native space according to the Destrieux Atlas (Destrieux et al., 2010; Fischl et al., 2004). Subsequently the corresponding parcellation in native space was registered to the b = 0 image from HARDI data of each subject and the following regions were extracted: insula (INS), mid-cingulate cortex (MCC), anterior cingulate cortex (ACC), medial prefrontal cortex (mPFC), primary somatosensory cortex (S1) and the nucleus accumbens (NAC). Similarly, the FreeSurfer subcortical segmentation was used to create masks and identify subregions for the amygdala/hippocampus (Iglesias et al., 2015; Saygin et al., 2017) and the thalamus (Iglesias et al., 2018). For the localization and segmentation of the hypothalamus (Spindler et al., 2020) and the cerebellum (Diedrichsen, 2006; Diedrichsen et al., 2009; Diedrichsen et al., 2011), masks in MNI space were used which were aligned to the b = 0 image from HARDI for each subject.

##### Brainstem regions

In contrast to subcortical and cortical regions, no sources are available to automatically select or segment regions in the brainstem. There are sources available providing atlases and masks for brainstem regions (Edlow et al., 2012; Keren et al., 2009; Pauli et al., 2018). However, respective ROIs in the brainstem in MNI space are difficult to warp to native space given that MNI space atlases are heavily weighted for cortical regions. Thus, the masks of the solitary nucleus (NTS), the nucleus ambiguus (NA), the motor nucleus of the vagus nerve (Xmn), the rostral ventrolateral medulla (RVLM), the caudal ventrolateral medulla (CVLM), the spinal nucleus of the trigeminal nerve (SpV), the parabrachial complex (PBC), the locus coeruleus (LC) and the periaqueductal grey (PAG) were created manually. These ROI masks are based on the localization relative to neuroanatomical landmarks and distinct contrast differences as indicated by different neuroanatomical atlases (Mai & Paxinos, 2012; Naidich et al., 2009; Nieuwenhuys et al., 2008; Paxinos & Huang, 2013) as well as the Brainstem wiki (http://fibratlas.univ-tours.fr/mediawiki/index.php), which is part of the Fibratlas Project that provides detailed descriptions on how to identify regions (Lechanoine et al., 2021). For the PBC, LC and PAG regions, additional masks in MNI space, provided by Edlow et al. (Edlow et al., 2012), were used to allocate the corresponding region for each subject. The slice editor of the MRview software was used to manually identify the regions slice by slice in the transversal plane. Additionally, coronal and sagittal views were used to identify neuroanatomical landmarks (e.g. superior/inferior colliculi, central canal, pontomedullary junction, pontomesencephalic junction etc.).

#### 2.2.5 | Selection of effective tractograms based on fractional anisotropy

In order to determine plausible direct white matter connections between the bilateral NTS and a specific set of ROIs as listed above, a conservative approach, using number of streamlines (NOS) and fractional anisotropy (FA), was applied. Given the exploratory nature of our study, we deemed NOS and FA, two common quantitative measures of tractography (F. Zhang et al., 2022), to be a good balance between allowing as much streamlines as possible and minimizing false positives. First, an FA map was calculated for each individual. Probabilistic white matter streamlines between the NTS and the respective ROIs were generated, and the underlying FA was subsequently sampled along the streamlines. Given the exploratory nature of this study, on a *subject level*, all tractograms with more than one streamline between the NTS and another ROI were analyzed (X ≥ 142 maximum possible connections given the number of 71 ROIs). Additionally, tractograms with a mean FA in the lowest 5th percentile were discarded. In other words, among all the available tractograms per subject, we used the lowest 5th percentile FA to exclude streamlines with mean FA lower than the threshold. The use of a percentile cutoff enabled us to eliminate tractograms with low FA, while taking individual differences in FA across subjects into account instead of using an arbitrary FA cut-off (e.g. 0.2) that has been traditionally employed. Last, on a *group level*, a sample number cut off was implemented, considering a specific pathway only if we could reliably reconstruct it for >4 subjects (i.e. >20% of *n* = 20). The mean FA value and streamline/seed ratios are indicated in Tables 1–4. The visualization of structural connectivity data was achieved by using CIRCOS (Krzywinski et al., 2009; Ziovas & Grigoriadou, 2008).

#### 2.2.6 | Resting state connectivity (RS)

##### Functional connectivity

After extracting the mean time courses, the functional connectivity (FC) of all ROIs to the NTS were examined, separately for the left and the right side. To establish FC, the absolute value of the Pearson correlation coefficient between any given pair of time courses was used. Thus, a total of 71 (ROIs) × 2 (bilateral NTS) correlations was calculated. For any pair of time courses with a high degree of freedom (TRs = 1200, *df* = 1198), an r1 ≥ 0.057 was deemed significant. All values lower than r1 were considered as zero for each individual. Afterwards, the group mean FC over all subjects for all the possible connections with the NTS was calculated. On the group level, we thresholded at r2 ≥ 0.098 (*p* < 0.05 for alpha = 0.05, Bonferroni corrected for the 71 possible connections) to get a list of ROIs that are significantly functionally connected to the left or the right NTS.

##### Granger causality

Only ROIs with significant FC to either NTS were explored further. However, given the confounders introduced by haemodynamic smoothing and the relatively low temporal resolution of fMRI, we will not place too much emphasis on individual relationships. Rather, the general trends observed will be described. To this end, as a proxy, the percentage of the sample showing significant Granger causality (GC) relationships with the NTS was used: (1) afferent connections, that is, from the NTS to the ROI could be X, or (2) efferent connections, that is, from the ROI to the NTS could be Y. The Matlab implementation of Granger causality analysis made public by Barnett and Seth was used for calculation (Barnett & Seth, 2014). An alpha correction was applied for the number of ROIs with significant FC to either NTS.

## 3 | RESULTS

### 3.1 | Systematic literature review

The systematic review of the MRI-based NTS literature identified a total of 137 PubMed entries. The literature was reviewed independently by two authors (JF and AMM), which identified 50 studies as suitable in the context of NTS connectivity. In total, 87 entries were excluded. The majority of manuscripts (*n* = 45) were excluded mostly due to an inappropriate allocation of the keyword ‘NTS’, as it is used as an abbreviation for various conditions and terms, for example, nano truck, nanopore targeted sequencing, non-typhoidal Salmonella, non-typhi Salmonella, normal prostatic gland tissue, neurotransmitters, nontreatment-seeking individuals with alcohol use disorder, nanotubes and so on. Additionally, case reports (*n* = 30) and review articles (*n* = 12) were excluded.

**FIGURE 1:**
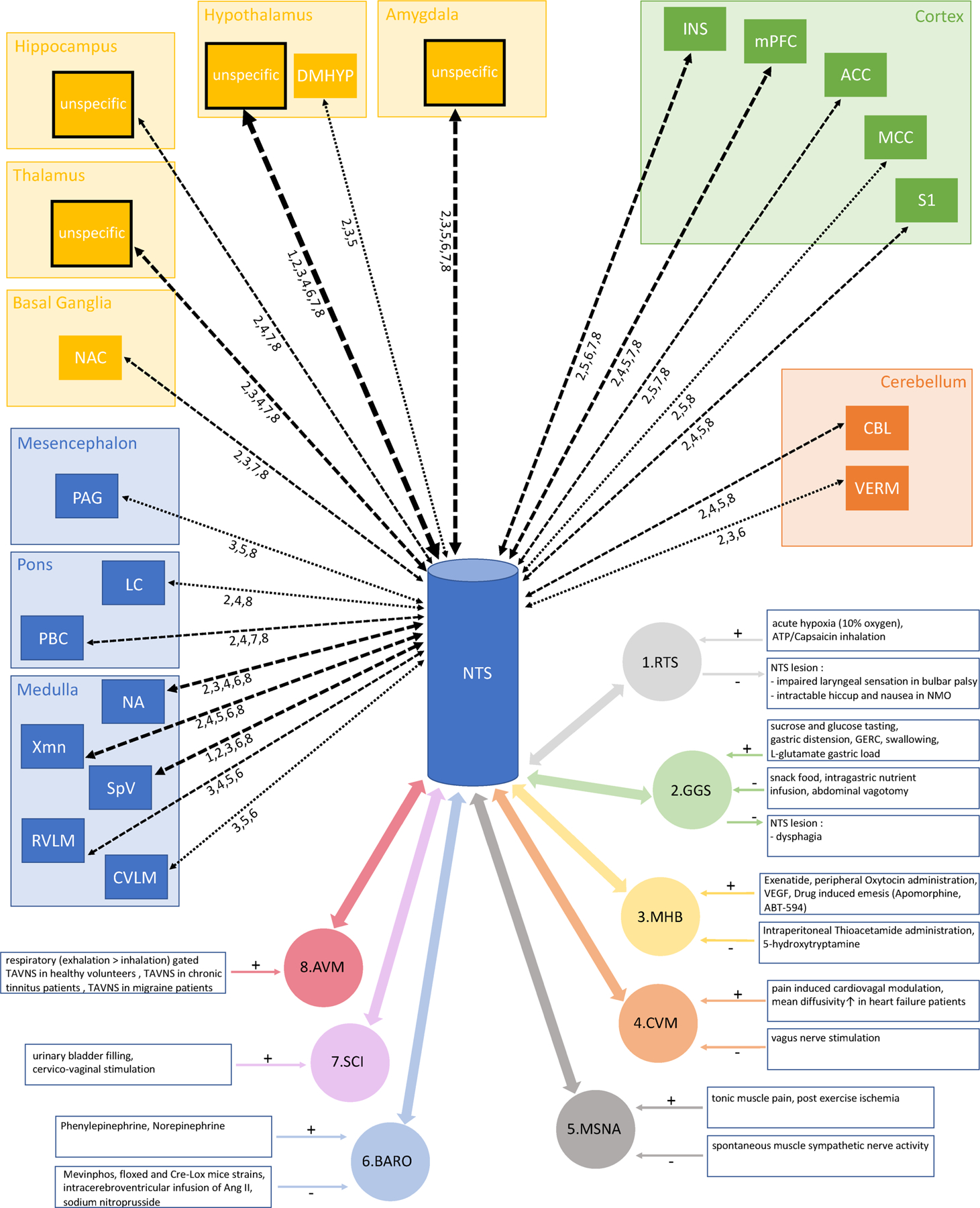
Conceptual map of NTS connectivity. Literature-based (Tables S1 and S3) connections from the NTS to ROIs in the brainstem (blue), the subcortex (yellow), the cortex (green) and the cerebellum (orange). Additionally, inhibitory (−) and activating (+) confounders towards each organ system are indicated in the blue boxes. ACC, anterior cingulate cortex; BG, basal ganglia; CBL, cerebellar hemisphere; CVLM, caudal ventrolateral medulla; DMHYP, dorsomedial hypothalamus; INS, insular cortex; LC, locus coeruleus; HIPPO, hippocampus; HYPO, hypothalamus; MCC, mid-cingulate cortex; mPFC, medial prefrontal cortex; NA, nucl. ambiguus; NAC, nucl. accumbens; PAG, periaqueductal grey; PBC, parabrachial complex; RVLM, rostral ventrolateral medulla; S1, primary somatosensory cortex; SpV, spinal nucl. of the V nerve; VERM, cerebellar vermis; Xmn, dorsal motor nucl. of the X nerve. Organ systems: 1. RTS = respiratory/tracheolaryngeal system; 2. GGS = gustatory/gastroesophageal system; 3. MHB = metabolism/hormonal balance; 4. CVM = cardiovagal modulation; 5. MSNA = muscle sympathetic nerve activity; 6. BARO = baroreflex; 7. SCI = spinal cord injury; 8. AVM = auriculovagal modulation

Based on the reviewed literature, the findings were classified into the organ system subcategories ‘respiratory/tracheolaryngeal system’, ‘gustatory/gastroesophageal system’, ‘metabolism/hormonal balance’, ‘auriculovagal modulation’, ‘cardiovagal modulation’, ‘morphological approaches’, ‘muscle sympathetic nerve activity’, ‘baroreflex’ and ‘spinal cord injury’ (see Table S1). The results of the systematic literature review were used to generate a conceptual map of NTS connectivity (see Figure 1). Those regions, indicating NTS involvement, at least in three different organ systems, were then selected for tractography and functional connectivity analyses. Additionally, a table listing all regions extracted from the systematic literature review as well as the involvement of each region within the organ systems is provided as a supplement (Table S3).

### 3.2 | Structural NTS connectivity

**FIGURE 2:**
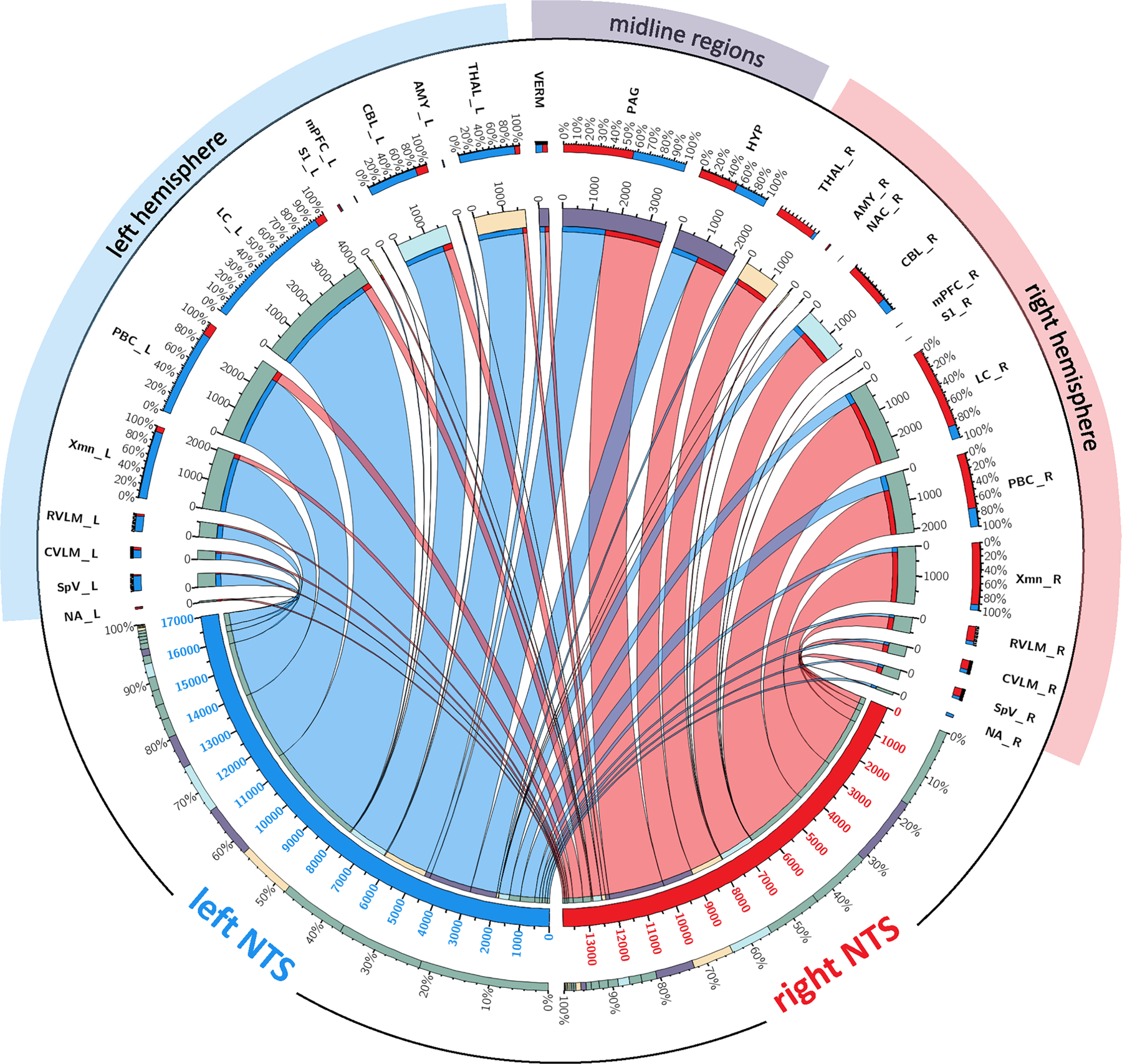
Circular map of structural connectivity. Normalized streamline/seed ratios from the left (blue) and right (red) NTS towards ipsilateral and contralateral brainstem (green), subcortical (yellow), cortical (black), cerebellar (turquoise) and midline (purple) regions. Notable is the general lateralization showing approximately 25% more connections from the left NTS. Note: Ratios = [streamline/seed ratios] × 10^4^. AMY, amygdala; CBL, cerebellar hemisphere; CVLM, caudal ventrolateral medulla; LC, locus coeruleus; HYP, hypothalamus; mPFC, medial prefrontal cortex; NA, nucl. ambiguus; NTS, nucl. tractus solitarius; PAG, periaqueductal grey; PBC, parabrachial complex; RVLM, rostral ventrolateral medulla; S1, primary somatosensory cortex; SpV, spinal nucl. of the V nerve; THAL, thalamus; VERM, cerebellar vermis; Xmn, dorsal motor nucl. of the X nerve; _L, left hemisphere; _R, right hemisphere

All effective white matter connections as well as connectivity strength in terms of (1) ipsilateral (i.e. right to right [rr] or left to left [ll]) versus contralateral (i.e. right to left [rl] or left to right [lr]) connections and (2) lateralization, that is, left-sided versus right-sided connections (i.e. ll-rr and lr-rl) are displayed in Tables 1–4. For visualization, a circular map of structural connectivity was created (see Figure 2).

### 3.3 | Brainstem

All brainstem regions preselected via literature research indicated a structural connection with the NTS. Overall, the ipsilateral connections on the right (rr) and left (ll) hemispheres were significantly stronger for the rostral ventrolateral medulla (RVLM; rr-rl: *p* < 0.001, ll-lr: *p* = 0.003), the spinal nucleus of the trigeminal nerve (SpV; rr-rl: *p* = 0.038, ll-lr: *p* < 0.001), the dorsal motor nucleus of the vagus nerve (Xmn; rr-rl: *p* < 0.001, ll-lr: *p* < 0.001), the parabrachial complex (PBC; rr-rl: *p* < 0.001, ll-lr: *p* < 0.001) and the locus coeruleus (LC; rr-rl: *p* = 0.001, ll-lr: *p* < 0.001). Conversely, only the contralateral right (rl) and left (lr) connections from the NTS to the contralateral nucleus ambiguus (NA; rr-rl: *p* < 0.001, ll-lr: *p* < 0.001) showed significantly stronger connections.

A lateralization (i.e. left vs. right) was always more prominent for left-sided connections (i.e. from the left NTS) as demonstrated by stronger left-sided ipsilateral (ll vs. rr: PBC: *p* = 0.012 and LC: *p* = 0.004) and contralateral (lr vs. rl: NA: *p* = 0.012; CVLM: *p* = 0.016; SpV: *p* = 0.007 and PBC: *p* = 0.047) connections. All *p*- and *z*-values are indicated in Table 1.

### 3.4 | Subcortical

Structural connections from the NTS were demonstrated towards the bilateral thalamus, hypothalamus and amygdala, but only for the right ipsilateral nucleus accumbens. There were no direct connections between the NTS and the hippocampus.

The thalamus (THAL; rr-rl: *p* < 0.001, ll-lr: *p* < 0.001) and the amygdala (AMY; rr-rl: *p* = 0.060, ll-lr: *p* = 0.016) showed more distinct ipsilateral then contralateral connections, consistent with the results from the brainstem. This finding is further corroborated by the respective subnuclei tracking, indicating stronger ipsilateral connections to the following thalamic subnuclei: intralaminar thalamic subnuclei group (THAL_IG; rr-rl: *p* < 0.001, ll-lr: *p* < 0.001), the medial thalamic subnuclei group (THAL_MG; rr-rl: *p* < 0.001, ll-lr: *p* < 0.001), the posterior thalamic subnuclei group (THAL_PG; rr-rl: *p* < 0.001, ll-lr: *p* < 0.001) and the ventral thalamic subnuclei group (THAL_VG; rr-rl: *p* < 0.001, ll-lr: *p* < 0.001). No structural ipsilateral or contralateral connections were found for the anterior and the lateral thalamic subnuclei group.

In line with above findings, the ipsilateral connections to the amygdala subnuclei included the accessory-basal nucleus (AMY_ABN; bilateral), the basal nucleus (AMY_BN; unilateral/right), the central nucleus (AMY_CEN; bilateral), the cortical nucleus (AMY_CON; bilateral), the corticoamygdaloid transition (AMY_COR; bilateral), the lateral nucleus (AMY_LN; bilateral) and the medial nucleus (AMY_MN; bilateral), whereas no contralateral connections except to the right medial nucleus could be detected. No structural ipsilateral or contralateral connections were found for the anterior-amygdaloid-area (AMY_AAA) and the paralaminar nucleus (AMY_PLN).

For the thalamic subnuclei, a lateralization towards stronger left-sided ipsilateral connections was demonstrated for the THAL_PG (ll-rr: *p* = 0.008) and a lateralization towards stronger right-sided contralateral connections for the THAL_MG (lr-rl: *p* = 0.015). Also, for the hypothalamus, a stronger right-sided connection towards the anterior superior hypothalamus (HYP_ant_sup; *p* = 0.004) was detected. All *p*- and *z*-values are indicated in Table 2.

### 3.5 | Cortex and cerebellum

Despite the literature review indicating connections to the anterior cingulate cortex (ACC), the mid-cingulate cortex (MCC), the insular cortex (INS), the primary somatosensory cortex (S1) and the medial prefrontal cortex (mPFC), we only found structural connections between the NTS and ipsilateral S1 and the ipsilateral and contralateral mPFC.

For the cerebellum, ipsilateral and contralateral structural connections were detected, showing significantly more ipsilateral than contralateral connections from the left NTS (ll-lr: *p* < 0.001). All *p*- and *z*-values are indicated in Tables 3 and 4.

### 3.3 | Functional and directional NTS connectivity

**FIGURE 3:**
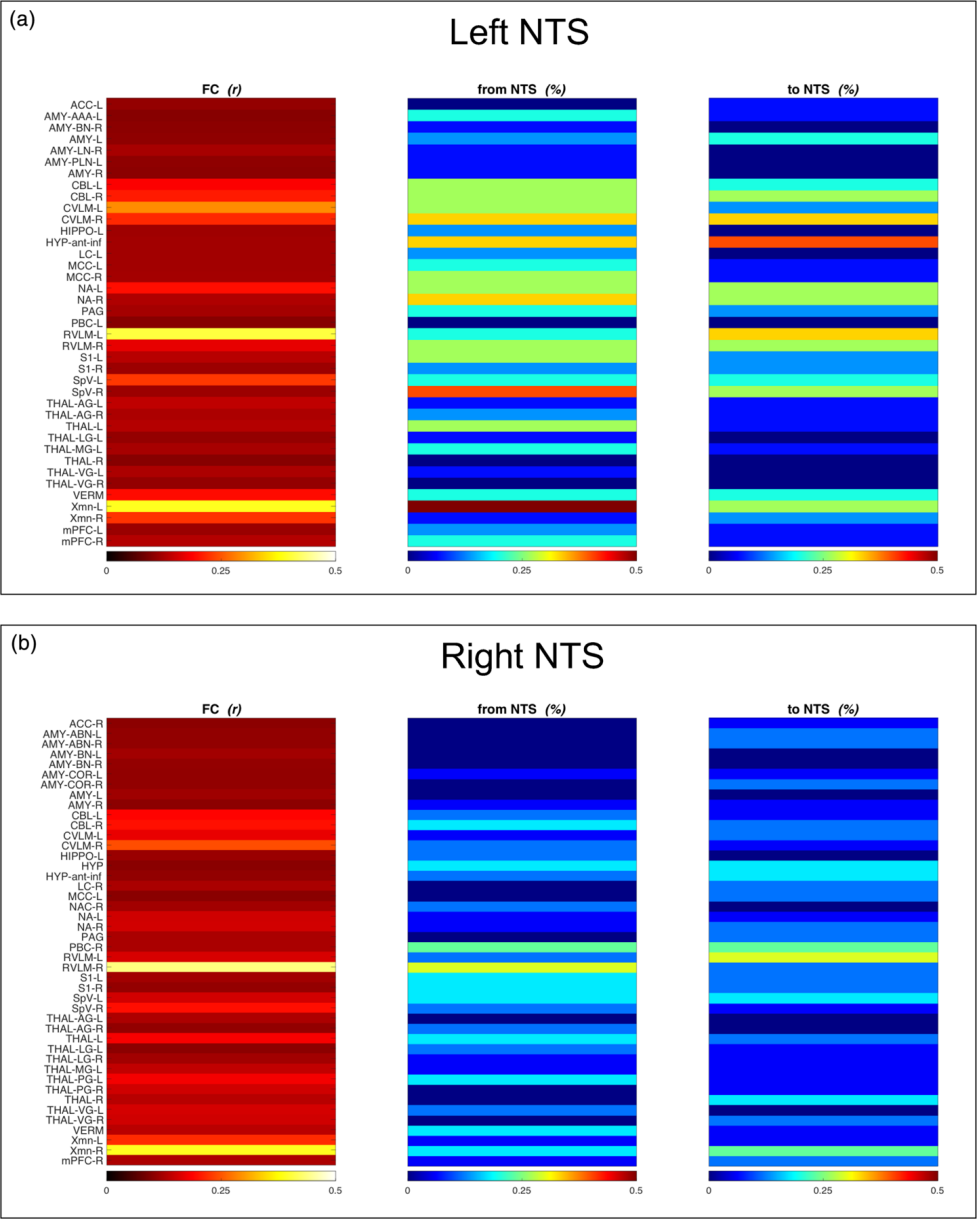
Functional and directional connectivity. Functional connectivity (FC, red tone) and the directional connectivity assessed via Granger-causality (GC, blue tone) for the left (a) and the right (b) NTS. For FC, the colour bars of each region indicate the correlation coefficients (*r*) as displayed by the colour spectrum. For GC, the colour bars of each region indicate the percentage (%) of subjects with directional connectivity, either from or to the NTS. Thereby, GC indicates if a region (i.e. the NTS) is more likely to receive or project to other CNS regions. The present results indicate that the NTS (left > right) relays more output (i.e. from the NTS to region X) than input (i.e. to the NTS from region X). Given, the role of the NTS as a sensory relay, these results suggest that the output reflects predominantly afferent signal processing from the NTS towards other brainstem regions and higher-order brain regions. ACC, anterior cingulate cortex; AMY, amygdala; AMY_AAA, anterior-amygdaloid-area; AMY_ABN, accessory-basal nucleus; AMY_BN, basal nucleus; AMY_COR, corticoamygdaloid nucleus; AMY_LN, lateral nucleus; AMY_PLN, paralaminar nucleus; CBL, cerebellar hemisphere; CVLM, caudal ventrolateral medulla; LC, locus coeruleus; HIPPO, hippocampus; HYP, hypothalamus; HYP_ant_inf, anterior inferior hypothalamic subnuclei; MCC, mid-cingulate cortex; mPFC, medial prefrontal cortex; NA, nucl. ambiguus; NAC, nucl. accumbens; NTS, nucl. tractus solitarius; PAG, periaqueductal grey; PBC, parabrachial complex; RVLM, rostral ventrolateral medulla; S1, primary somatosensory cortex; SpV, spinal nucl. of the V nerve; THAL, thalamus; THAL_AG, anterior thalamic group; THAL_LG, lateral thalamic group; THAL_MG, medial thalamic group; THAL_PG, posterior thalamic group; THAL_VG, ventral thalamic group; VERM, cerebellar vermis; Xmn, dorsal motor nucl. of the X nerve; _L, left hemisphere; _R, right hemisphere

The functional connectivity (FC) was calculated for all regions, previously selected via literature review [i.e. 71 (ROIs) × 2 (bilateral NTS)]. A significant FC was shown for the left NTS in 39 regions and for the right NTS in 44 regions. The Granger causality (GC) analysis indicated that a qualitatively higher percentage of subjects have a lateralization of signal processing from/to the left NTS. Moreover, the GC indicates a directionality pattern, suggesting that the NTS is predominantly involved in afferent signal processing (i.e. from the NTS towards upstream CNS regions). A visualization of this trend is provided in Figure 3.

## 4 | DISCUSSION

We investigated the structural and functional connectivity of the NTS in vivo in humans, based on a systematic review of the literature. Our study confirms previous results from animal models and provides novel aspects on NTS connectivity and integration in humans.

To our knowledge, this is one of the first investigations in human NTS research, based on a prior systematic literature research. Our results support feasibility of studying structural connections between the NTS, a small structure in the brain stem, and several regions in the CNS. We found lateralized connections, with a predominantly left-sided affinity of the NTS. Furthermore, functional connectivity analyses showed consistent results with those from tractography (i.e. directly connected regions have significant correlation of time courses. Last, we also investigated plausible directionality of such connections and found mostly afferent connections from the NTS (left > right).

Below, we discuss implications of our findings with regard to various organ systems. We also address caveats to our findings in the context of the approach used to define the connectivity of the NTS within the brainstem, subcortical and cortical regions and the specifics of connectivity as defined by tractography.

### 4.1 | Animal–human translation

The interpretation of our approach was based on the following: (1) details of specific connectivity reported for animals and (2) the known functional aspects of the NTS defined in preclinical models and evaluated in fMRI studies in humans. Thereby, we acknowledge various aspects of NTS research and address critical considerations within translational research:

(1) In the past, morphological and physiological NTS research has been mostly conducted in rats (de de Jong & Palkovits, 1976; Doba & Reis, 1973; Mtui et al., 1993; Mtui et al., 1995; Ricardo & Koh, 1978; Ruggiero et al., 1998; Ruyle et al., 2018), in addition to other mammals such as cats (Ciriello & Calaresu, 1980; Morest, 1967), guinea pigs (Allen, 2004) or rabbits (Douglas et al., 1956). In this context, a review of the NTS literature utilizing tract tracing in rodents and cats (Altschuler et al., 1989; Blessing et al., 1981; Ciriello & Caverson, 1986; Ciriello et al., 1981; Craig, 1995; Finley & Katz, 1992; Housley et al., 1987; Kalia & Mesulam, 1980; Kapp et al., 1985; Loewy et al., 1981; McDougall et al., 2017; Ricardo & Koh, 1978; Ross et al., 1981; Shin et al., 2008; Shipley, 1982; van der van der Kooy et al., 1984; Viltart et al., 2006; Wallach & Loewy, 1980) indicated consistency with regard to the NTS connectivity within the brainstem (i.e. regions identified by animal research: NA, PAG, DMX, LC, VLM, PBC, SpV). However, with regard to connectivity towards subcortical (i.e. regions identified by animal research: paraventricular nucleus of the hypothalamus, medial preoptic nucleus of the hypothalamus, periventricular nucleus of the thalamus, bed nucleus of the striae terminalis, AMYce) or cortical regions (i.e. regions identified by animal research: anterior and posterior insular cortex, infra- and prelimbic medial prefrontal cortex) less research was conducted, and some tracts were not preserved. For example, projections from the insular cortex to the NTS in rodents (Kapp et al., 1985; Shipley, 1982) were not confirmed in the human. Due to the relative paucity of data available with regard to NTS connectivity towards cortical and subcortical regions, the presented study extends previous knowledge by adding a systematic tracking to previously identify key regions and their subnuclei, that is, within the thalamus, the hypothalamus and the amygdala.

Fewer approaches to study NTS pathways have been made in primates (Beckstead et al., 1980; Kinney, 1978; Norgren, 1990), which however also suggested species-specific variations in comparison with other mammals (Ruggiero et al., 2000). For example, only in rats a strong cocaine-addiction related projection from the NTS to the periventricular thalamus was found (Brown et al., 1992; Ruggiero et al., 1998). Other than that, cats (Ciriello & Calaresu, 1980) and rats (Ruggiero et al., 1998; Ter Horst & Postema, 1997) do not exhibit a direct pathway from the NTS to discriminative subnuclei of the thalamus (i.e. ventral posteromedial thalamic nucleus [VMP], ventral posterolateral thalamic nucleus [VPL]), which conversely has been shown in primates (Beckstead et al., 1980). The latter result is supported by our study indicating a structural and functional connectivity to the ventral thalamic nuclei group (=THAL_VG), which includes the VMP and the VPL. Moreover, it was argued that in the cat visceral ascending pathways from the NTS are interrupted in the bulbar reticular formation and the dorsal tegmental nucleus and thus are limited to the brainstem (Morest, 1967; Warren Cottle & Calaresu, 1975); however, our findings show distinct connectivity beyond the regions they describe.

In general, the lack of translation between animal and human research is a known issue that has been addressed in a meta-analysis approach that included the most highly cited articles (>500 citations) of the seven leading scientific journals (i.e. Science, Nature, Cell etc.) by citation impact factor (Hackam & Redelmeier, 2006). Although the assumption that this research represents the most accurate proceedings, with higher chances of being tested in clinical trials (Ioannidis, 2005), only 37% were replicated, 18% were contradicted and 45% remained untested in human randomized trials (Hackam & Redelmeier, 2006). Even more concerning was a report that only 6% of animal studies translate to human research (Shuler, 2017). This translational gap between basic and clinical research, also referred to as ‘the valley of death’ (A. A. Seyhan, 2019b), certainly has multifactorial sources with the species-specific neuronal pathways representing only one component. However, in light of the current findings, we would like to emphasize that NTS connectivity towards primitive brainstem structures seems to be preserved across different species, whereas NTS connectivity towards higher-order cortical or subcortical regions may substantially differ between rodents and primates. Apart from species-specific issues, there are other problems such as the ‘butterfly-effect’ that pertains to a chaotic behaviour of preclinical animal models or the ‘two cultures’ problem that refers to different clinical and preclinical methodologies (A. Seyhan, 2019a). With regard to these critical considerations, we think that the present approach is a necessary step to shed light on human NTS connectivity. Moreover, we acknowledge some issues raised above, by analysing publicly available highly standardized datasets from subjects of the HCP database, which fosters transparency of protocol information and reproducibility.

(2) In humans, most NTS research was conducted via task fMRI research. This however predominantly followed functional aspects and disregarded structural connectivity properties of the NTS. Also, the fact that those results were obtained under different circumstances (i.e. scanners, protocols, in healthy vs. patient populations) limits its informative value.

Nevertheless, our conceptual map of regions (which also included MRI-based NTS research in animals) provided a hypothesis for potential NTS connectivity that we address systematically in the underlying study.

### 4.2 | Lateralization aspects of NTS connectivity

There are physiological data in animals available suggesting lateralization of function and morphology. However, as pointed out previously, these data cannot be translated to humans unrestrictedly but rather should act as supporting evidence.

In terms of NTS lateralization, there are several options why a structural or functional connectivity can be pronounced towards one side of the body. We will use data provided on baroreflex function to discuss principles of lateralization, as this pathway and its components have been thoroughly analysed in animals in the past.

The baroreflex is mediated via baroreceptors (i.e. afferent glossopharyngeal and vagal nerve fibre terminals), which are localized in the aortic arch and carotid sinus. A decrease of arterial blood pressure reduces afferent impulses from the vagal nerve which project to the NTS and further to the NA (Ciriello & Calaresu, 1981; Schwartz & De Ferrari, 2011). Subsequently, this increases efferent sympathetic nerve activity via an excitatory relay to the CVLM and an inhibitory relay to the RVLM. Thus, in hypotensive conditions, the presympathetic neurons in the RVLM function via a disinhibition mechanism (Freeman, 2008). In the event of a hypertensive state, the NTS excites vasodepressor neurons in the NA and the Xmn, additionally to an inhibition of vasopressor neurons in the RVLM. In the face of respectively increasing and decreasing blood pressure, the baroreflex thereby adjusts the cardiac output via parasympathetic and sympathetic activity (Wallbach & Koziolek, 2018).

First, the peripheral distribution of receptors projecting to the NTS may be lateralized. For example, in terms of aortic arch innervation, which mediate baro- and chemoreceptive information, more pronounced sensory innervation towards the left aortic arch was suggested (Z. Cheng et al., 1997a). In addition, the vagal afferent innervation of the left and the right atrium of the heart was investigated in rats, indicating a larger size of atrial ganglionic neurons (Z. Cheng et al., 1997b). In line with these morphological findings, the stimulation of left aortic baroreceptors exhibited a greater baroreflex response (i.e. decrease of blood pressure) than the stimulation of the right-sided receptors (Salman et al., 2020).

Second, a lateralization may follow the functional demand of the effector organ. As previously indicated, the role of the NTS primarily pertains to the integration of sensory signals. The NTS projects directly to brainstem nuclei such as the NA or the RVLM that carry visceroefferent fibres. Thus, lateralization in such pathways may reflect innervation properties of effector organs. Different studies in rodents and primates indicated that the cortical innervation of the myocardium is predominantly mediated via right-sided efferents (Chuang et al., 2004; Ter Horst & Postema, 1997). It was reported that in monkeys a retrograde staining from the sinoatrial node (SA) labelled 75% neurons in the right NA and only 25% in the left NA (Chuang et al., 2004). These observations may reflect the distribution of our neuronal pathway observations, indicating a stronger connection from the left NTS towards the right NA (lr-rl: *p* = 0.012). Additionally, also a trend towards a dominant connection from the left NTS to the right RVLM may reflect this notion (lr-rl: *p* = 0.053). However, in rats, a comparison of left and right NA projections towards cardiac ganglia was not as distinct (Zixi Cheng & Powley, 2000).

### 4.3 | Functional implications: The NTS—An integrator of viscero-sensory information

Apart from studying the NTS connectivity in the human, our findings are discussed within clinical and preclinical observations of selected organ systems. Thereby, future research within different areas of the autonomic nervous system should be stimulated.

#### 4.3.1 | Muscle sympathetic nerve activity (MSNA)

The MSNA reflex is a direct measure of the efferent sympathetic nerve activity. The reflex is thought to regulate vasomotor activity through vascular smooth muscles within skeletal muscles. In the past, the lateralization of efferent sympathetic nerve activity has been addressed by measuring MSNA in left and right peroneal nerves in humans (Diedrich et al., 2009; Sundlöf & Wallin, 1977; Sverrisdóttir et al., 1998). It was claimed that at rest, there is a MSNA lateralization towards the right, which however is abolished due to baroreflex activation (Diedrich et al., 2009). As discussed previously, the demand of the effector organ may determine pathway lateralization, that is, in this case, a dominant right-sided MSNA at rest may require a dominant right-sided baroreflex response to abolish lateralization.

The role of the NTS within the MSNA mainly pertains to the integration of muscle afferent input via the spinoreticular tracts that project to the RVLM and CVLM, which alter the baroreceptor set point to allow blood pressure adaption during exercise (Barman & Yates, 2017; Degtyarenko & Kaufman, 2002; Masuda et al., 1992; Potts, 2001, 2006; Stornetta et al., 1989; Wilson et al., 2002). In the past, spontaneous fluctuations in MSNA correlated to activity changes in brainstem regions mediating baroreflex function, such as the NTS, the RVLM and the CVLM (Macefield & Henderson, 2010). More specifically, it was shown that an increase in MSNA (i.e. in response to spontaneous arterial hypotension) induced a decrease in fMRI signal intensity in the CVLM and NTS and an increase in fMRI signal intensity in the RVLM (Henderson et al., 2012). The present study indicates a more frequent functional connectivity from the bilateral NTS towards the right RVLM, and the tractography reveals a trend towards a more dominant contralateral connectivity from the left NTS towards the right RVLM (lr-rl: *p* = 0.053). Similar, the connectivity from the bilateral NTS to the right CVLM is indicated more frequently, and the tractography indicates a significantly stronger contralateral connectivity from the left NTS towards the right CVLM (lr-rl: *p* = 0.016). Both results are in line with a potentially lateralized baroreflex response to abolish lateralized MSNA.

Transferring these considerations to pathophysiological observations in the clinical routine, the complex regional pain syndrome (CRPS) is a debilitating unilateral neuralgia that among other pathophysiological features often shows an increase of sympathetic nerve activity in the affected limb. In terms of lateralization, a study indicated that in children the right foot is more frequently affected (Abu-Arafeh & Abu-Arafeh, 2016) and that in chronic CRPS patients, the left/right ratio was at 12/21 (Wittayer et al., 2018). Additionally, the latter study found that the perception as well as ownership of a rubber-hand was more severely impaired in right-sided CRPS patients (Wittayer et al., 2018). Given these clinical observations, the physiological lateralization of the MSNA towards the right might add to the understanding of increased vulnerability and disease severity towards CRPS development on the right body side.

#### 4.3.2 | Gustatory/oesophageal system

A fundamental process that pertains to food ingestion, independent of the composition of the nutrient, is the act of swallowing. The process of swallowing is a complex interaction of sensor and motor function. All sensory input from the cranial nerves V, VII, IX and X converge at the NTS (Jean, 2001).

Besides the local processing, within the brainstem, the NTS further projects to numerous cerebral regions (e.g. thalamus, basal ganglia, the supplemental motor area, anterior cingulate cortex, cerebellum etc.) (Martin et al., 2001; K. Mosier & Bereznaya, 2001; Rangarathnam et al., 2014; Suzuki et al., 2003; Zald & Pardo, 1999). Interestingly, different studies suggested a lateralization of cortical function in swallowing with a dominance in the left hemisphere (Harris et al., 2005; K. M. Mosier et al., 1999; Suzuki et al., 2003). Additionally, the role of the cerebellum especially of the left cerebellar hemisphere has been suggested collectively (Rangarathnam et al., 2014; Sasegbon & Hamdy, 2021). These findings are supported by our results indicating a stronger structural connection from the left NTS towards the left than the right cerebellar hemisphere (CBL; ll-lr: *p* < 0.001) as well as a trend between the left and right ipsilateral NTS-CBL connection (ll-rr: *p* = 0.064). The notion of left and right hemispheric differences in swallowing also translates into clinical observations such as that left-hemispheric strokes prolong the prepharyngeal response time and right-hemispheric strokes severe pharyngeal dysfunction (i.e. reduced laryngeal elevation, lingual discoordination) (Daniels et al., 1999; Kwon et al., 2005). The observation, in particular towards left hemispheric pathology, is a possible consequence of a dysfunctional sensory processing which presumably is more dominant via left-sided NTS afferents. These considerations may also have implications for logopaedic post-stroke rehabilitation.

In terms of food ingestion, a study in rodents suggested only a left-sided activation during the tasting task and a bilateral NTS activation during gastric distention (Roelofs et al., 2020). Moreover, another rodent study aimed to study conditioned flavour preference mediated via the vagus nerve suggesting higher T-values in the left > right NTS consistently in the sham and common hepatic branch vagotomies. Additionally, ‘incomplete’ abdominal branch vagotomies with intact hepatic branches, completely abolished the BOLD signal of the right but not the left NTS (Uematsu et al., 2010). These findings further underpin the relevance of sensory processing via pathways of the left NTS.

#### 4.3.3 | Metabolism/hormonal balance

Diabetes is among the most common metabolic disorders with approximately 50.1 million people (13.1%) living in the North American and Caribbean region (Huang et al., 2018) and with obesity being the major risk factor for type 2 diabetes (Pinkney, 2002). Thus, the focus of this discussion will be within this framework.

Our results support the relevance of the NTS in food intake regulation, by indicating a functional connectivity between both left > right NTS to the inferior anterior hypothalamic group (HYPO_inf_ant). The HYPO_inf_ant group which includes the ventromedial nucleus of the hypothalamus (VMH) has been claimed as the satiety centre; however, to date, its specific role is not entirely clear (King, 2006). The fact that the VMH inherits a large number of glucoresponsive neurons (Anand et al., 1964; Oomura, 1973; Oomura et al., 1969; Song et al., 2001) underpins its relevance for its metabolic function. Together with previous notions from the gustatory/oesophageal system, the NTS may add important sensory information with regard to gastric dilatation and food preference that may guide the decision of food intake.

### 4.4. | Tractography approach to connectivity analysis of a small structure

Finally, we would like to address our approach for an adequate ROI localization within the brainstem. The NTS is a small structure, and in order to capture it, we followed the process defined in the methods section. There are potential pitfalls accompanying this approach, including capturing brain outside the specific region of interest (ROI). Still, the rigor and reproducibility of our approach for a correct ROI allocation within the brainstem is supported by the following.

**FIGURE 4:**
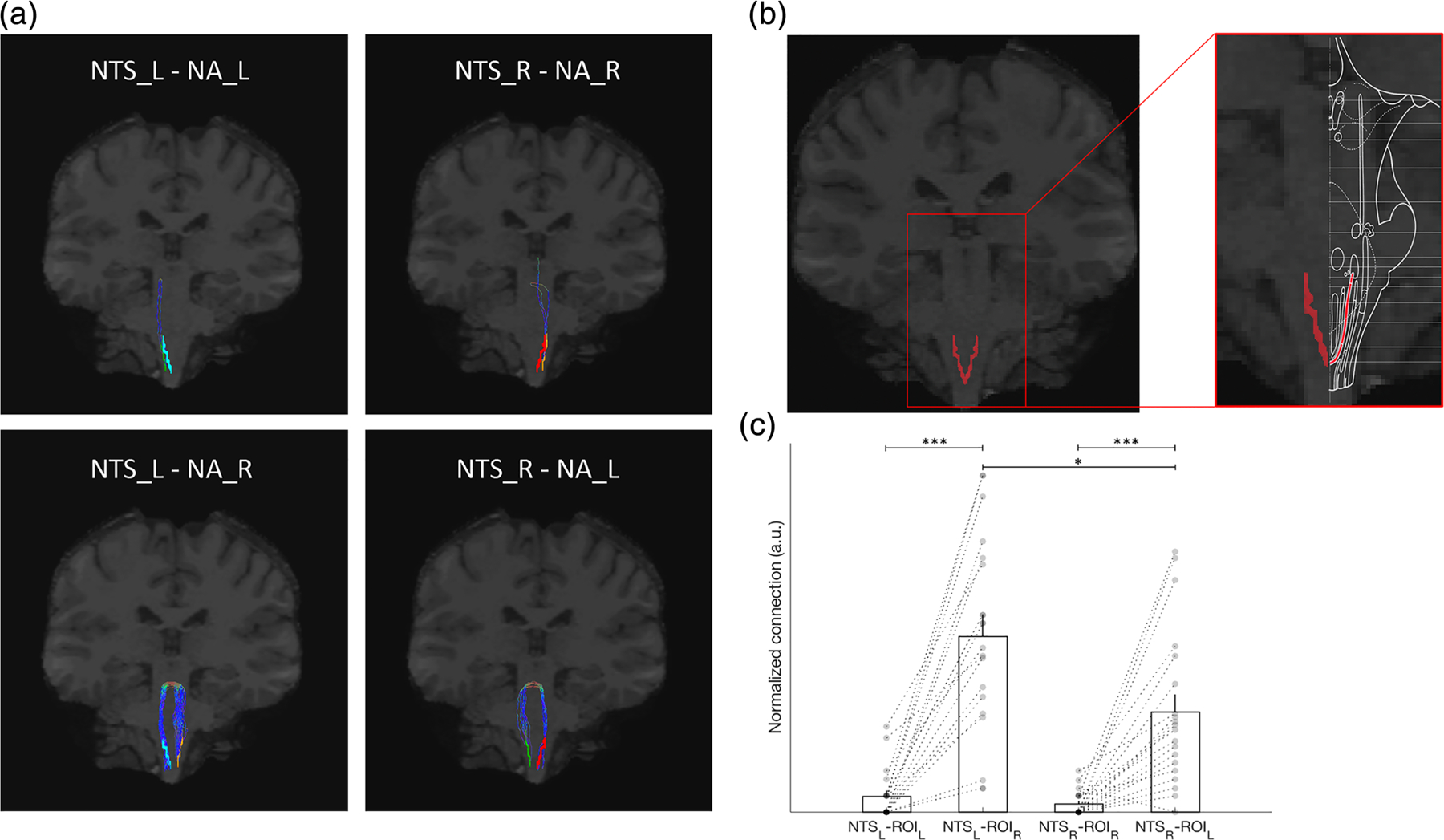
Localization of the NTS. (a) Displayed is the distinct connectivity pattern of the NTS-NA structural connectivity, which indicates a predominance towards contralateral connections. (b) We present the bilateral NTS ROIs (left) as well as a brainstem atlas overlay from the corresponding region provided by Duvernoy’s atlas (right) (Naidich et al., 2009). (c) Normalized streamline/seed ratios of the NTS-NA connection indicate a stronger contralateral connection over all subjects. Light blue = NTSl; red = NTSr; green = NAl; orange = NAr

First, our results with regard to the selected streamlines are very consistent across all subjects, with low standard errors of the mean (see Tables 1–4). Second, we provide the NTS ROI of one representative individual (subj. ID 101107 in the HCP database), which corresponds to the location of the NTS as provided by Duvernoy’s atlas (Naidich et al., 2009) (see Figure 4b). Third, the streamline selection follows distinct patterns, of which some have been previously described in animal models. An example for a distinct pattern that would be very difficult to reproduce in the event of a ROI dislocation is the dominant contralateral connection from the NTS to the nucleus ambiguus (NA, see Figure 4c) (Naidich et al., 2009). We have provided the visualization of a tractography sample (subj. ID 101107), which exhibits a streamline selection representative for the group mean (i.e. subj/group: NTSl-NAl [2/2.05], NTSl-NAr [25/21.5], NTSr-NAr [2/1.3], NTSr-NAl [9/12.3]; see Figure 4a). It is notable that most nuclei in very close proximity to the NA exhibit dominant ipsilateral connections, such as the rostral ventrolateral medulla (RVLM). Thus, if the NA were incorrectly placed, the outlined pattern would not have been visible.

## 5 | LIMITATIONS

This study has a few limitations that include: (1) *Sex*. Due to the random selection of patients the skewness of male versus female participants needs to be considered in terms of potential sexual dimorphism. (2) *Small size of nucleus*. In terms of spatial resolution, the HARDI data acquired via the HPC project, account for a resolution of 1.25 mm. The NTS diameter ranges from 1 to 2 mm in the human (Lechanoine et al., 2021); thus, the spatial resolution certainly needs to be taken into account as it may diminish accuracy of structure location. Although, the identification of structures within the brainstem proves difficult several other attempts to identify brainstem structures at corresponding field strength (3 tesla) and similar spatial resolution were successfully conducted in the past, suggesting feasibility of the applied method (Habas & Cabanis, 2007; Mueller, 2020; Straub et al., 2019). The selection of functionally distinct subregions within the NTS or other brainstem regions would add importantly to the current knowledge. However, given the previously mentioned limitations of this pilot study, the authors decided to refrain from a further subsegmentation. (3) *Sample size*. This pilot study aims to generate further hypothesis of the NTS’ integration within the central nervous system; thus, more specific NTS research should apply large group tracking. (4) *Subject comfort/vigilance*. The scanning was not supervised by the authors; thus, comfortable positioning or vigilance of the subjects was not under our control. (5) *Lateralization*. Due to methodological limitations a bilateral tracking to the hypothalamus and the periaqueductal grey could not be conducted; thus, information of lateralization aspects is limited.

## 6 | CONCLUSIONS

Within different domains, signs of lateralization have been observed in clinical and preclinical approaches. However, only a minority of NTS literature tried to address these lateralization findings in humans.

Here, we utilized a systematic approach to investigate the role of the human NTS, within different autonomic functions. Moreover, we addressed several issues within NTS research, such as the discordance between animal and human research, and thus added important data on white matter connectivity as well as functional connectivity.

The current thesis presented in the underlying study may allow future work to determine a distinction within the different domains (i.e. organ systems) of the autonomic nervous system.

## Supplementary Material

3 Supplementary Files in a Word Document

Additional supporting information can be found online in the Supporting Information section at the end of this article.

## Figures and Tables

**TABLE 1:** Structural DTI connectivity of brainstem regions

Label	NTS_L		NTS_R		NTS_R-X_R vs. NTS_L-X_L	NTS_R-X_R vs. NTS_R-X_L	NTS_L-X_L vs. NTS_L-X_R	NTS_L-X_R vs. NTS_R-X_L
	Mean [± SEM]	FA [± SEM]	Mean [± SEM]	FA [± SEM]				
BRAINSTEM								
NTS_L	-	-	1.60 [± 0.22]	0.44 [± 0.01]	-	-	-	*p* = 0.765
NTS_R	1.63 [± 0.23]	0.44 [± 0.02]	-	-	-	-	-	*Z* = −0.299
NA_L	0.08 [± 0.03]	0.47 [± 0.00]	0.49 [± 0.08]	0.49 [± 0.01]	*p* = 0.454	***p* < 0.001**	***p* < 0.001**	***p* = 0.012**
NA_R	0.86 [± 0.11]	0.49 [± 0.01]	0.04 [± 0.01]	0.47 [± 0.02]	*Z* = −0.749	***Z* = −4.881**	***Z* = −5.126**	***Z* = 2.503**
CVLM_L	2.36 [± 0.58]	0.44 [± 0.01]	0.66 [± 0.08]	0.48 [± 0.01]	*p* = 0.882	*p* = 0.239	*p* = 0.607	***p* = 0.016**
CVLM_R	1.05 [± 0.12]	0.49 [± 0.01]	2.55 [± 0.66]	0.44 [± 0.01]	*Z* = 0.149	*Z* = 1.177	*Z* = 0.514	***Z* = 2.408**
RVLM_L	4.44 [± 1.34]	0.44 [± 0.01]	0.60 [± 0.10]	0.50 [± 0.01]	*p* = 0.871	***p* < 0.001**	***p* = 0.003**	*p* = 0.053
RVLM_R	0.95 [± 0.13]	0.50 [± 0.01]	4.32 [± 0.99]	0.43 [± 0.01]	*Z* = 0.162	***Z* = 3.761**	***Z* = 2.990**	*Z* = 1.935
SpV_L	4.32 [± 1.01]	0.44 [± 0.01]	0.41 [± 0.08]	0.50 [± 0.01]	*p* = 0.053	***p* = 0.038**	***p* < 0.001**	***p* = 0.007**
SpV_R	0.86 [± 0.15]	0.50 [± 0.01]	2.29 [± 0.61]	0.44 [± 0.01]	*Z* = −1.935	***Z* = 2.071**	***Z* = 3.476**	***Z* = 2.719**
Xmn_L	19.41 [± 1.22]	0.45 [± 0.02]	1.59 [± 0.24]	0.48 [± 0.02]	*p* = 0.675	***p* < 0.001**	***p* < 0.001**	*p* = 0.684
Xmn_R	1.68 [± 0.38]	0.48 [± 0.02]	17.71 [± 1.73]	0.45 [± 0.01]	*Z* = −0.419	***Z* = 4.371**	***Z* = 5.399**	*Z* = −0.407
PBC_L	24.80 [± 2.75]	0.45 [± 0.01]	3.56 [± 0.44]	0.48 [± 0.01]	***p* = 0.012**	***p* < 0.001**	***p* < 0.001**	***p* = 0.047**
PBC_R	5.38 [± 0.65]	0.49 [± 0.01]	15.85 [± 2.09]	0.44 [± 0.01]	***Z* = −2.502**	***Z* = 5.167**	***Z* = 5.288**	***Z* = 1.988**
LC_L	37.41 [± 3.41]	0.42 [± 0.01]	2.95 [± 0.32]	0.46 [± 0.01]	***p* = 0.004**	***p* = 0.001**	***p* < 0.001**	*p* = 0.130
LC_R	3.97 [± 0.45]	0.48 [± 0.01]	23.25 [± 3.36]	0.42 [± 0.01]	***Z* = −2.884**	***Z* = 3.234**	***Z* = 4.856**	*Z* = 1.515
PAG	15.34 [± 2.39]	0.41 [± 0.00]	20.08 [± 3.45]	0.43 [± 0.01]	*p* = 0.364	-	-	-
					*Z* = 0.908	-	-	-

*Note*: Displayed is the mean [± standard error of the mean] of selected streamline/seed ratios and the fractional anisotropy (FA) for each brainstem region (X), respectively for the tracking from the left (NTS_L) or right (NTS_R) NTS. Statistical analysis: Wilcoxon signed rank test. Bold items indicate significance (*p* < 0.05).

Abbreviations: CVLM, caudal ventrolateral medulla; LC, locus coeruleus; NA, nucl. ambiguus; NTS, nucl. tractus solitarius; PAG, periaqueductal grey; PBC, parabrachial complex; RVLM, rostral ventrolateral medulla; SpV, spinal nucl. of the V nerve; Xmn, dorsal motor nucl. of the X nerve; _L, left hemisphere; _R, right hemisphere.

**TABLE 2:** Structural DTI connectivity of subcortical regions

Label	NTS_L		NTS_R		NTS_R-X_R vs. NTS_L-X_L	NTS_R-X_R vs. NTS_R-X_L	NTS_L-X_L vs. NTS_L-X_R	NTS_L-X_R vs. NTS_R-X_L
	Mean [± SEM]	FA [± SEM]	Mean [± SEM]	FA [± SEM]				
Subcortex								
THAL_L	16.07 [± 2.42]	0.45 [± 0.01]	1.42 [± 0.37]	0.44 [± 0.01]	*p* = 0.250	***p* < 0.001**	***p* < 0.001**	*p* = 0.256
THAL_R	0.81 [± 0.11]	0.44 [± 0.01]	11.76 [± 1.45]	0.44 [± 0.01]	*Z* = −1.150	***Z* = 5.235**	***Z* = 5.397**	*Z* = −1.137
THAL_AG_L			-	-	-	-	-	-
THAL_AG_R			-	-	-	-	-	-
THAL_IG_L	9.69 [± 1.30]	0.46 [± 0.01]	0.49 [± 0.12]	0.44 [± 0.01]	*p* = 0.234	***p* < 0.001**	***p* < 0.001**	*p* = 0.092
THAL_IG_R	0.18 [± 0.03]	0.44 [± 0.01]	7.40 [± 1.00]	0.46 [± 0.00]	*Z* = −1.190	***Z* = 5.344**	***Z* = 5.403**	*Z* = −1.686
THAL_LG_L			-	-				
THAL_LG_R			-	-				
THAL_MG_L	6.53 [± 1.32]	0.43 [± 0.01]	0.44 [± 0.14]	0.39 [± 0.01]	*p* = 0.860	***p* < 0.001**	***p* < 0.001**	***p* = 0.015**
THAL_MG_R	0.07 [± 0.03]	0.38 [± 0.01]	6.22 [± 1.30]	0.42 [± 0.00]	*Z* = −0.176	***Z* = 4.911**	***Z* = 5.228**	***Z* = −2.444**
THAL_PG_L	1.81 [± 1.11]	0.44 [± 0.01]	0.03 [± 0.02]	0.46 [± 0.03]	***p* = 0.008**	***p* ≤ 0.001**	***p* ≤ 0.001**	*p* = 0.985
THAL_PG_R	0.02 [± 0.01]	0.44 [± 0.01]	0.38 [± 0.14]	0.45 [± 0.01]	***Z* = −2.668**	***Z* = 3.718**	***Z* = 5.236**	*Z* = −0.019
THAL_VG_L	8.49 [± 2.07]	0.46 [± 0.01]	1.15 [± 0.37]	0.45 [± 0.01]	*p* = 0.070	***p* < 0.001**	***p* < 0.001**	*p* = 0.465
THAL_VG_R	0.59 [± 0.11]	0.45 [± 0.01]	4.72 [± 0.56]	0.45 [± 0.01]	*Z* = −1.812	***Z* = 4.775**	***Z* = 5.344**	*Z* = −0.731
AMY_L	0.29 [± 0.08]	0.44 [± 0.01]	0.04 [± 0.02]	0.44 [± 0.01]	*p* = 0.490	*p* = 0.060	***p* = 0.016**	*p* = 0.764
AMY_R	0.06 [± 0.03]	0.44 [± 0.01]	0.35 [± 0.19]	0.43 [± 0.01]	*Z* = −0.690	*Z* = 1.879	***Z* = 2.414**	*Z* = 0.300
AMY_AAA_L			-	-	-	-	-	-
AMY_AAA_R			-	-	-	-	-	-
AMY_ABN_L	0.12 [± 0.03]	0.44 [± 0.01]	-	-	*p* = 1.000	-	-	-
AMY_ABN_R			0.18 [± 0.09]	0.42 [± 0.01]	*Z* = 0.000	-	-	-
AMY_BN_L			-	-	-	-	-	-
AMY_BN_R			0.07 [± 0.05]	0.42 [± 0.01]	-	-	-	-
AMY_CEN_L	0.10 [± 0.05]	0.42 [± 0.01]	-	-	*p* = 0.744	-	-	-
AMY_CEN_R			0.07 [± 0.03]	0.42 [± 0.01]	*Z* = −0.327	-	-	-
AMY_CON_L	0.12 [± 0.03]	0.44 [± 0.01]	-	-	*p* = 0.977	-	-	-
AMY_CON_R			0.23 [± 0.11]	0.43 [± 0.01]	*Z* = −0.029	-	-	-
AMY_COR_L	0.04 [± 0.02]	0.44 [± 0.01]	-	-	*p* = 0.631	-	-	-
AMY_COR_R		-	0.11 [± 0.05]	0.42 [± 0.01]	*Z* = 0.481	-	-	-
AMY_LN_L	0.03 [± 0.01]	0.42 [± 0.00]	-	-	*p* = 0.897	-	-	-
AMY_LN_R		-	0.07 [± 0.05]	0.41 [± 0.01]	*Z* = 0.130	-	-	-
AMY_MN_L	0.18 [± 0.06]	0.43 [± 0.01]	-	-	*p* = 0.351	-	***p* = 0.046**	-
AMY_MN_R	0.02 [± 0.01]	0.43 [± 0.02]	0.19 [± 0.14]	0.43 [± 0.01]	*Z* = −0.934	-	***Z* = 2.000**	-
AMY_PLN_L		-	-	-	-	-	-	-
AMY_PLN_R		-	-	-	-	-	-	-
NAC_L		-	-	-	-	-	-	-
NAC_R		-	0.03 [± 0.01]	0.44 [± 0.01]	-	-	-	-
HIPPO_L		-	-	-	-	-	-	-
HIPPO_R		-	-	-	-	-	-	-
HYP	9.49 [± 1.34]	0.41 [± 0.01]	11.09 [± 1.70]	0.40 [± 0.01]	*p* = 0.525	-	-	-
					*Z* = 0.636	-	-	-
HYP_ant_inf	4.18 [± 1.19]	0.39 [± 0.01]	3.82 [± 1.34]	0.38 [± 0.01]	*p* = 0.483	-	-	-
					*Z* = −0.701	-	-	-
HYP_ant_sup	0.44 [± 0.16]	0.40 [± 0.01]	2.45 [± 0.71]	0.40 [± 0.01]	***p* = 0.004**	-	-	-
					***Z* = 2.876**	-	-	-
HYP_int	3.84 [± 0.54]	0.42 [± 0.01]	3.30 [± 0.60]	0.42 [± 0.01]	*p* = 0.365	-	-	-
					*Z* = −0.906	-	-	-
HYP_post	7.94 [± 1.23]	0.41 [± 0.01]	8.67 [± 1.24]	0.41 [± 0.01]	*p* = 0.695	-	-	-
					*Z* = 0.392	-	-	-

*Note*: Displayed is the mean [± standard error of the mean] of selected streamline/seed ratios and the fractional anisotropy (FA) for each subcortical region (X), respectively, for the tracking from the left (NTS_L) or right (NTS_R) NTS. Statistical analysis: Wilcoxon signed rank test. Bold items indicate significance (*p* < 0.05).

Abbreviations: AMY, amygdala; AMY_AAA, anterior-amygdaloid-area; AMY_ABN, accessory-basal nucleus; AMY_BN, basal nucleus; AMY_CEN, central nucleus; AMY_CON, cortical nucleus; AMY_COR, corticoamygdaloid nucleus; AMY_LN, lateral nucleus; AMY_MN, medial nucleus; AMY_PLN, paralaminar nucleus; DMHYP, dorsomedial hypothalamus; HIPPO, hippocampus; HYP, hypothalamus; HYP_ant_inf, anterior inferior hypothalamic subnuclei; HYP_ant_sup, anterior superior hypothalamic subnuclei; HYP_int-intermediate hypothalamic nuclei; HYP_post, posterior hypothalamic nuclei; NAC, nucl. accumbens; THAL, thalamus; THAL_AG, anterior thalamic group; THAL_IG, intralaminar thalamic group; THAL_LG, lateral thalamic group; THAL_MG, medial thalamic group; THAL_PG, posterior thalamic group; THAL_VG, ventral thalamic group; _L, left hemisphere; _R, right hemisphere.

**TABLE 3:** Structural DTI connectivity of cortical regions

Label	NTS_L		NTS_R		NTS_R-X_R vs. NTS_L-X_L	NTS_R-X_R vs. NTS_R-X_L	NTS_L-X_L vs. NTS_L-X_R	NTS_L-X_R vs. NTS_R-X_L
	Mean [± SEM]	FA [± SEM]	Mean [± SEM]	FA [± SEM]				
Cortex								
ACC_L	-	-	-	-	-	-	-	-
ACC_R	-	-	-	-	-	-	-	-
MCC_L	-	-	-	-	-	-	-	-
MCC_R	-	-	-	-	-	-	-	-
INS_L	-	-	-	-	-	-	-	-
INS_R	-	-	-	-	-	-	-	-
S1_L	0.26 [± 0.18]	0.49 [± 0.01]	-	-	*p* = 0.182	-	-	-
S1_R	-	-	0.04 [± 0.01]	0.53 [± 0.02]	*Z* = −1.334	-	-	-
mPFC_L	0.17 [± 0.06]	0.43 [± 0.01]	0.06 [± 0.04]	0.41 [± 0.02]	*p* = 0.409	*p* = 0.249	-	-
mPFC_R	-	-	0.12 [± 0.05]	0.41 [± 0.01]	*Z* = −0.825	*Z* = 1.152	-	-

*Note*: Displayed is the mean [± standard error of the mean] of selected streamline/seed ratios and the fractional anisotropy (FA) for each cortical region (X), respectively, for the tracking from the left (NTS_L) or right (NTS_R) NTS. Statistical analysis: Wilcoxon signed rank test. Bold items indicate significance (*p* < 0.05).

Abbreviations: ACC, anterior cingulate cortex; INS, insular cortex; MCC, mid-cingulate cortex; mPFC, medial prefrontal cortex; S1, primary somatosensory cortex; _L, left hemisphere; _R, right hemisphere.

**TABLE 4:** Structural DTI connectivity of cerebellar regions

Label	NTS_L		NTS_R		NTS_R-X_R vs. NTS_L-X_L	NTS_R-X_R vs. NTS_R-X_L	NTS_L-X_L vs. NTS_L-X_R	NTS_L-X_R vs. NTS_R-X_L
	Mean [± SEM]	FA [± SEM]	Mean [± SEM]	FA [± SEM]				
Cerebellum								
CBL_L	14.47 [± 2.20]	0.48 [± 0.01]	3.26 [± 0.33]	0.50 [± 0.01]	p = 0.064	p = 0.317	**p < 0.001**	p = 0.457
CBL_R	3.89 [± 0.49]	0.51 [± 0.01]	12.36 [± 4.01]	0.46 [± 0.01]	Z = −1.853	Z = 1.001	**Z = 4.112**	Z = 0.744
VERM	1.93 [± 0.37]	0.49 [± 0.01]	1.42 [± 0.34]	0.48 [± 0.01]	p = 0.279	-	-	-
					Z = −1.082	-	-	-

Note: Displayed is the mean [± standard error of the mean] of selected streamline/seed ratios and the fractional anisotropy (FA) for each cerebellar region (X), respectively, for the tracking from the left (NTS_L) or right (NTS_R) NTS. Statistical analysis: Wilcoxon signed rank test. Bold items indicate significance (p < 0.05).

Abbreviations: CBL, cerebellar hemisphere; VERM, cerebellar vermis; _L, left hemispher; _R, right hemisphere.

## Data Availability

Data were provided by the Human Connectome Project, WU-Minn Consortium (Principal Investigators: David Van Essen and Kamil Ugurbil; 1U54MH091657) funded by the 16 NIH Institutes and Centers that support the NIH Blueprint for Neuroscience Research and by the McDonnell Center for Systems Neuroscience at Washington University. The data that support the findings of this study are openly available from the Human Connectome Project database at https://db.humanconnectome.org/, of the WU-MINN HCP Data release (1200 subjects).

## Abbreviations:

ACC: anterior cingulate cortex
AMY: amygdala
AMY_AAA: anterior-amygdaloid-area
AMY_ABN: accessory-basal nucleus
AMY_BN: basal nucleus
AMY_CEN: central nucleus
AMY_CON: cortical nucleus
AMY_COR: corticoamygdaloid nucleus
AMY_LN: lateral nucleus
AMY_MN: medial nucleus
AMY_PLN: paralaminar nucleus
CBL: cerebellar hemisphere
CVLM: caudal ventrolateral medulla
DMHYP: dorsomedial hypothalamus
HIPPO: hippocampus
HYP: hypothalamus
HYP_ant_inf: anterior inferior hypothalamic subnuclei
HYP_ant_sup: anterior superior hypothalamic subnuclei
HYP_int: intermediate hypothalamic nuclei
HYP_post: posterior hypothalamic nuclei
INS: insular cortex
LC: locus coeruleus
MCC: mid-cingulate cortex
mPFC: medial prefrontal cortex
MRI: magnetic resonance imaging
NA: nucl. ambiguous
NAC: nucl. accumbens
NTS: nucl. tractus solitarius
ROI: region of interest
PAG: periaqueductal gray
PBC: parabrachial complex
RVLM: rostral ventrolateral medulla
S1: primary somatosensory cortex
SpV: spinal nucl. of the V nerve
THAL: thalamus
THAL_AG: anterior thalamic group
THAL_IG: intralaminar thalamic group
THAL_LG: lateral thalamic group
THAL_MG: medial thalamic group
THAL_PG: posterior thalamic group
THAL_VG: ventral thalamic group
VERM: cerebellar vermis
Xmn: dorsal motor nucl. of the X nerve
