# Supplementary material for: The solitary nucleus connectivity to key autonomic regions in humans
Source: Eur J Neurosci. Author manuscript; Available in PMC 2026 Feb 19. (PMC12918705; doi:10.1111/ejn.15691)
Supplement: 3 Supplementary Files in a Word Document [file NIHMS2107818-supplement-3_Supplementary_Files_in_a_Word_Document.docx]

**Supplementary Files**

**Supl. Table 1. MRI-based research of the NTS.**

|  | | | | |
| --- | --- | --- | --- | --- |
| MRI-based Research of the NTS | | | | |
| Author | **Subjects** | **Technique** | **Intervention** | **Primary outcome** |
| Respiratory/Tracheolaryngeal System | | | | |
| (Gerlach et al., 2021) | H, n=12 | fMRI | episodes of 10% oxygen supply | Acute hypoxia indicates altered functional connectivity of the NTS with medullary and hypothalamic regions |
| (Farrell et al., 2020) | H, n=20 | 3T, fMRI | Inhalation of nebulized ATP and capsaicin solution | Airway nodose pathways project centrally to the NTS |
| (Bautista, Leech, Mazzone, & Farrell, 2019) | H, n=15 | 3T, fMRI | Capsaicin inhalation for airway irritation | Capsaicin inhalation showed activation in the NTS, paratrigeminal nucl, spinal trigeminal nucl. and tract, VLM and midline raphe |
| (Ishibashi & Fujishima, 2012) | H, n=15 | MRI | Patients with lateral medullary infarction | A lesion of the NTS induces impaired laryngeal sensation in bulbar palsy patients. |
| (Misu, Fujihara, Nakashima, Sato, & Itoyama, 2005) | H, n=47 | 0.5T/1.5T, MRI | Intractable hiccup and nausea (IHN) in:  - neuromyelitis optica (NMO)  - multiple sclerosis (MS) | IHN was found in none of the MS cases but in 17% of NMO cases. In 6 cases lesions involved the pericanal region, the area postrema and the NTS. |
| Gustatory/Gastroesophageal System | | | | |
| (Roelofs et al., 2020) | A, n=13 | 9.4T, fMRI | Sucrose tasting and gastric distention | Gastric distention > sucrose tasting induce NTS activation |
| (Frank-Podlech et al., 2019) | H, n=11 | 3T, rsMRI | Prior ingestion  Post ingestion:  low-fat vs. high-fat meal | After ingestion, functional connectivity between NTS and reward/gustatory areas was lower. |
| (Z. Chen et al., 2019) | A, n=60 | 7T, Manganese-enhanced MRI | Induction of Gastroesophageal reflux-gated cough (GERC) due to HCl and citric acid perfusion in the lower esophagus. | GERC induced Fos expression in the NTS, DMX, paratrigeminal nucl., intermediate reticular nucl. |
| (Hoogeveen, Dalenberg, Renken, ter Horst, & Lorist, 2015) | H, n=24 | 3T, fMRI | Tasting of sweet, sour, salty and bitter in young  (mean age 23 y) and old (mean age 65 y) healthy volunteers | No age effect in taste information, based on absence of difference in activation of NTS and ventral posteromedial nucl. of the thalamus. |
| (Tsurugizawa & Uneyama, 2014) | A, n=18 | 4.7T, fMRI | Intragastrical infused glucose vs. saccharin | Both glucose and saccharin increase BOLD signal in the NTS and the insular cortex. |
| (Mihai, von Bohlen Und Halbach, & Lotze, 2013) | H, n=21 | 3T, fMRI | Swallowing vs. occlusion (temporomandibular joint movement) | Brainstem activation for swallowing in the sensory nucleus of the trigeminal nerve and the NTS. Brainstem activation for occlusion in the trigeminal nerve. |
| (Hoch, Kreitz, Gaffling, Pischetsrieder, & Hess, 2013) | A, n=8 | 7T, Manganese-enhanced MRI | Standard chow vs. snack food | Snack food induced a deactivation of the NTS as compared to animals fed with standard chow. |
| (Min, Tuor, Koopmans, & Chelikani, 2011) | A, n=26 | 9.4T, MRI | Influence of intragastric nutrient infusion of saline, mixed nutrient or protein. | Intragastric nutrient infusion reduced the BOLD fMRI signal in homeostatic (hypothalamus, NTS) and non-homeostatic centers (thalmus, hippocampus, caudate, putamen, cerebral cortex, cerebellum) |
| (Tsurugizawa, Uematsu, Uneyama, & Torii, 2011) | A, n=14 | 4.7T, fMRI | Intragastric load of monosodium L-glutamate (MSG) and inosine monophosphate (IMP) | Intragastric load of MSG or IMP increased the BOLD signal in the NTS, lateral hypothalamus, and insular cortex. The BOLD responses in the hippocampus and orbital cortex were associated with NTS activation. |
| (Uematsu et al., 2010) | A, n=40 | 4.7T, fMRI | Intragastric load of monosodium L-glutamate (MSG)  + sham  + total abdominal branch vagotomie (TVX)  + common hepatic branch vagotomie (HVX)  + total abdominal branch vagotomie, hepatic branch intact (TVXh) | BOLD signal was increased after MSG administration in the NTS, in the Sham, HVX and TVXh group.  Amygdala, hippocampus, lat. Hypothalamus were activated in the Sham and HVX group. |
| (Tsurugizawa, Uematsu, Uneyama, & Torii, 2010) | A, n=24 | 4.7T, fMRI | Visceral nutrient signal (L-Glutamate) in rats:  -Isoflurane anesthesia  -Alpha-chloralose anesthesia  -conscious | BOLD signal intensity in the NTS increased in all three conditions. |
| (Prosiegel, Höling, Heintze, Wagner-Sonntag, & Wiseman, 2005) | H, n=43 | 1.5T, MRI | Dysphagic patients with:  - unilateral paresis of the vagal nerve (PVN)  - Avellis’ Syndrome (AS)  - Wallenberg’s Syndrome (WS)  - posterior fossa tumor (PFT)  - cerebellar hemorrhage (CH) | In humans, the dorsomedial central pattern generators (CPGs) for swallowing are situated in the NTS, the reticular formation (RF). The dorsomedial CPGs are superior to the ventrolateral CPGs (NA/near RF) with regard to swallowing relevance. |
| (Topolovec, Gati, Menon, Shoemaker, & Cechetto, 2004) | H, n=8 | 4T, fMRI | - General visceral activation (GVA):  isometric hand-grip, maximal inspiration, Valsalva maneuver  - Special visceral activation (SVA):  sucrose administration | In all GVA tasks NTS and the parabrachial nucl. activation was observed. The activation of the NTS during sucrose administration was more rostral than in other GVA tasks. During maximal inspiration and Valsalva maneuver PAG activation was observed. During maximal inspiration and isometric handgrip task raphe activation was observed. |
| Metabolism/Hormonal Balance | | | | |
| (Coveleskie et al., 2017) | H, n=19 | 3T, fMRI | 5ugs Exenatide in females with normal BMI and obesity | Exanatide increased functional connectivity (FC) between the left NTS and the left thalamus and hypothalamus in the obese group, as well as fostered correlation of hunger scores and NTS FC in all groups. |
| (C. Y. Tsai, Su, Chan, & Chan, 2017) | A, n=172 | 9.4T, MRI, DWI, DTI | Hepatic Encephalopathy induced by intraperitoneal thioacetamide administration | DTI indicated a nitrosative stress- induced disruption of NTI and RVLM connectivity, together with a cessation of the sympathetic motor tone. |
| (Ferris et al., 2015) | A, n=46 | 7T, fMRI | peripheral oxytocin administration  central oxytocin administration | Peripheral oxytocin administration activated the olfactory bulb, cerebellar regions and among other regions of the ANS the NTS. |
| (C. Y. Tsai et al., 2015) | A, n=12 | 9.4T, MRI, DTI | Fetal liver kinase-1 (Flk-1) deficient mice | VEGF produces a tonic facilitation of the cardiovagal baroreflex via fetal liver kinase-1 in the NTS. |
| (Chin et al., 2006) | A, n=35 | 4.7T, pharmacological MRI | Drug induced emesis:  -apomorphine  -ABT-594 | A dose dependent relationship between rCBV changes and percent activated area within the NTS and area postrema was found. |
| (Henderson et al., 2002) | A, n=9 | 4.7T, fMRI | 5-hydroxytryptamine i.v.  - low dose 10 ug/kg  - high dose 20-30 ug/kg | High doses induced a rapid decrease of activation in the intermediate portion of the NTS, the CVLM, RVLM and fastigial nucl. of the cerebellum. Slightly delayed phasic decline appeared in the dentate and interpositus nuclei and dorsolateral pons. Late-developing decreases in the NTS, parapyramidal region, PAG, spinal trigeminal nucleus, inferior olivary nucleus, cerebellar vermis, fastigial nucleus. Amygdala und hypothalamus showed late and prolonged signal increase. |
| Auriculovagal Modulation | | | | |
| (Sclocco et al., 2020) | H, n=30 | 3T, fMRI | Respiratory gated transcutaneous auricular vagus nerve stimulation (RAVANS) at 2, 10, 25, 100 Hz | Stimulus frequency of RAVANS effects the fMRI (BOLD) response of the NTS |
| (Y. Zhang et al., 2019) | H, n=26 | 3T, fMRI, rsMRI | transcutaneous auricular vagus nerve stimulation (taVNS) at 1HZ in migraine patients. | taVNS decreases fMRI signals within the default mode network (e.g. NTS) |
| (Sclocco et al., 2019) | H, n=16 | 7T, fMRI | Respiratory gated transcutaneous auricular vagus nerve stimulation (RAVANS) | RAVANS during exhalation enhanced NTS targeting |
| (Yakunina, Kim, & Nam, 2018) | H, n=36 | 3T, fMRI | Transcutaneus vagus nerve stimulation (tVNS) (inner tragus, cymba conchae and earlobe (sham)) in patients with tinnitus | tVNS (i.t., c.c.) showed activations in locus coeruleus and NTS in tinnitus patients, but deactivation in multiple limbic and auditory regions. |
| (Garcia et al., 2017) | H, n=32 | 3T, fMRI | Respiratory gated transcutaneous auricular vagus nerve stimulation (RAVANS) in episodic migraine patients | Exhalatory RAVANS activated the ipsilateral NTS. NTS connectivity was increased to ant. insular and anterior midcingulate cortex. Increased connectivity was inversely correlated to time until next migraine attack |
| (Frangos & Komisaruk, 2017) | H, n=13 | 3T, fMRI | -sternocleidomastoid transcutaneous electrical stimulation (SCM, control condition)  -non-invasive vagus nerve transcutaneous electrical stimulation (nVNS, experimental condition) | Compared to SCM, the nVNS condition showed significant activation in the NTS, parabrachial area, primary somatosensory and insular cortex, basal ganglia and fronto-cortical regions. |
| (Yakunina, Kim, & Nam, 2017) | H, n=37 | 3T, fMRI | Different locations of vagus nerve stimulation:  (i) inner tragus, (ii) inferoposterior wall of the ear canal,  (iii) cymba conchae, (iv) earlob (=sham) | Stimulation of the cymba conchae showed the strongest activation in the NTS and locus coeruleus. |
| (Frangos, Ellrich, & Komisaruk, 2015) | H, n=12 | 3T, fMRI | Vagus nerve stimulation of the cymba conchae and the earlobe | Cymba conchae stimulations produced significant activations in the ipsilateral NTS, bilateral spinal trigeminal nucleus, dorsal raphe, locus coeruleus, contralateral parabrachial area, amygdala, and nucleus accumbens, Deactivations in the thalamus and hypothalamus. |
| Cardiovagal Modulation | | | | |
| (Sclocco et al., 2016) | H, n=11 | 7T, fMRI | Pain induced cardiovagal modulation (high frequency – heart rate variability, HF-HRV) | The most prominent cardiovagal reduction of HF-HRF within the first two minutes, showed activity changes in the DMX, NTS and locus coeruleus in addition to the RVLM, ventral nucl. reticularis/nucl. ambiguus, pontine nuclei. |
| (Woo et al., 2015) | H, n=42 | 3T, fMRI | Heart failure patients vs. healthy controls | Heart failure subjects showed increased mean diffusity in limbic, basal-ganglia, thalamic, frontal and cerebellar regions and the NTS. |
| (Reyt et al., 2010) | A, n=8 | 4.7T, fMRI | Vagus nerve stimulation | VNS showed deactivations in the NTS and closely connected structures such as the parabrachial nucl., locus coeruleus and hippocampus, cerebellum, prefrontal cortex and retrosplenial cortex. |
| Muscle Sympathetic Nerve Activity (MSNA) | | | | |
| (Macefield & Henderson, 2019) | H, n=37 | 3T, fMRI | muscle sympathetic nerve activity (MSNA):  tonic muscle pain (physiological)  obstructive sleep apnoe  (pathological) | Physiological muscle sympathetic nerve activity was positively related to BOLD signal response in the NTS |
| (Sander, Macefield, & Henderson, 2010) | H, n=17 | 3T, fMRI | - submaximal static handgrip  - post exercise ischemia (PEI) | PEI induced a bilateral BOLD signal increase in the NTS and the RVLM and thus supports that the metaboreflex is mediated by the medulla. |
| (Macefield & Henderson, 2010) | H, n=8 | 3T, fMRI | Spontaneous Muscle Sympathetic Nerve Activity (MSNA) | Increases in MSNA were associated with bilateral RVLM activity increase and decrease in NTS and CVLM.  Additional increase in activity was shown in the caudal pressor area, medullary raphe and DMX |
| Baroreflex | | | | |
| (C.-Y. Tsai, Poon, Chan, & Chan, 2019) | A, n=44 | 9.4T, DTI | Baroreflex functionality:  + Norepinephrine (physiological)  + Mevinphos intoxication (pathological) | Robust connectivity between the NTS and the rostral ventrolateral medulla is disrupted or severed due to baroreflex alterations. |
| (Gerlach et al., 2019) | H, n=11 | 3T, fMRI | Phenylepinephrin i.v. | Phenylepinephrine elicited a pressor response followed by a baroreflex-response which elicited signal changes in the NTS, CVLM, RVLM, nucl. ambiguus, nucleus raphe obscurus and other brainstem nuclei. |
| (C. Y. Tsai, Poon, Chen, & Chan, 2017) | H, n=69 | 9.4T, MRI, DTI | Cre-Lox and floxed mice strains | Anomalous baroreflex functionality in floxed and cre-lox mice as corroborated by a reduced NTS and RVLM and diminished sympathetic vasomotor tone and cardiac vagal baroreflex. |
| (C. Y. Tsai et al., 2013) | A, n=7 | 9.4T, MRI, DTI | Intracerebroventricular infusion of Angiotensin II | An augmented level of superoxide level in the NTS suppresses the cardiovagal baroreflex by disrupting the connectivity between NTS and nucl. ambiguus during neurogenic hypertension. |
| (Henderson et al., 2004) | A, n=7 | 4.7T, fMRI | Baroreceptor reflex activation:  - Pressor challenge (phenylephrine)  - Depressor challenge (sodium nitroprusside) | Depressor challenges produced signal decreases in the NTS, RVLM, CVLM, cerebellar vermis, inferior olive, dorsolateral pons and right insula. Signal increases were observed in the amygdala and hypothalamus. Pressor challenges elicited smaller increases in medullary regions, dorsolateral pons, the right insula and decreases in the hypothalamus. Both responses were lateralized. |
| Spinal Cord Injury | | | | |
| (Krhut et al., 2017) | H, n=12 | 3T, fMRI | Urinary bladder filling in patients with complete spinal cord injury | Significant activation during urinary bladder filling in the NTS, parabrachial nucl., hypothalamus, thalamus, amygdala, insular lobe, ant. cingulate gyrus, prefrontal cortex. Activations of NTS and parabrachial nucl. correlated with bladder sensations. |
| (Komisaruk et al., 2004) | H, n=5 | 1.5T, fMRI | Spinal cord injury at T10 or above:  - vaginal-cervical self-stimulation (CSS) | CSS showed activation of the superior region of the NTS. Brain regions activated during orgasm included hypothalamic paraventricular nucleus, medial amygdala, anterior cingulate, frontal, parietal and insular cortices, cerebellum. |
| (Whipple & Komisaruk, 2002) | H, n=3 | PET-MRI | Complete spinal cord injury above T10:  - vaginal-cervical self-stimulation (CSS) | CSS increased activity in the region of the NTS |
| Morphological Approaches | | | | |
| (Singh et al., 2019) | H, n=12 | 7T, DTI | none | Probabilistic template of the viscero-sensory-motor nuclei complex which among others includes the NTS, vagus nerve nucl. and hypoglossal nucl. |
| (Priovoulos, Poser, Ivanov, Verhey, & Jacobs, 2019) | H, n=20 | 7T | none | NTS mask based on magnetization transfer contrast manipulation |
| (Nishikawa et al., 2014) | H, n=40 | 3T, MRI  3DAC-PROPELLER | none | Identification of 5 tracts (corticospinal tr., medial lemniscus, medial longitudinal fasciculus, central tegmental and spinothalamic tr.) and 8 nuclei (oculomotor and trochlear nucl., spinal trigeminal, abducens, facial, vestibular, hypoglossal, prepositus, and solitary nucl.) |
| (Komisaruk et al., 2002) | H, n=7 | 1.5T, fMRI | Localization of brainstem nuclei via different tasks:  - brushing the face (sensory trigeminal n.)  - left right eye movement (abducens n.)  - smiling and lip puckering (facial n.)  - pushing tongue against hard palate (hypoglossal n.)  - swallowing (nucl. ambiguus)  - tasting sweet-sour-salty-bitter mixture (NTS)  - finger tapping (nucl. cuneatus)  - tongue movement to activate strap muscles (C1-C3) | The CN nuclei of the pons, medulla and other nuclei of the lower brainstem and cervical spinal cord can be identified via fMRI in combination with specific sensory stimulation or motor tasks. |

Displayed is an overview of the original work that has been used to create the conceptual map. The conceptual map is based on the findings from the 8 different organ systems: “Respiratory/Tracheolaryngeal System”; “Gustatory/Gastroesophageal System”; “Metabolism/Hormonal Balance”; “Cardiovagal Modulation”; “Muscle Sympathetic Nerve Activity”; “Baroreflex”; “Spinal cord injury”; “Auriculovagal Modulation”. Additionally, original work from morphological approaches is provided.

**Key:** n = number of participants; H = humans; A = animals

**Supl. Table 2. Characterization of HCP subjects.**

| **Subject ID** | **Age** | **Gender** | **Acquisition site** |
| --- | --- | --- | --- |
| 101107 | 22-25 | M | WASH U-HCP3T |
| 101309 | 26-30 | M | WASH U-HCP3T |
| 103414 | 22-25 | F | WASH U-HCP3T |
| 103818 | 31-35 | F | WASH U-HCP3T |
| 105014 | 26-30 | F | WASH U-HCP3T |
| 105115 | 31-35 | M | WASH U-HCP3T |
| 111312 | 31-35 | F | WASH U-HCP3T |
| 113619 | 31-35 | F | WASH U-HCP3T |
| 115320 | 31-35 | F | WASH U-HCP3T |
| 118528 | 26-30 | F | WASH U-HCP3T |
| 118932 | 26-30 | M | WASH U-HCP3T |
| 123925 | 26-30 | F | WASH U-HCP3T |
| 124422 | 31-35 | F | WASH U-HCP3T |
| 125525 | 31-35 | F | WASH U-HCP3T |
| 126325 | 26-30 | F | WASH U-HCP3T |
| 127630 | 22-25 | F | WASH U-HCP3T |
| 128127 | 26-30 | F | WASH U-HCP3T |
| 128632 | 31-35 | F | WASH U-HCP3T |
| 131217 | 26-30 | F | WASH U-HCP3T |
| 133928 | 26-30 | M | WASH U-HCP3T |

Indicated are the subject identifier numbers of the subjects analyzed. Additionally, the age range and gender are indicated. The image acquisition of all subjects was generated and made available by the WU-Minn Consortium of the Human Connectom Project. All subjects were scanned at a customized Siemens 3T “Connectom Skyra” at Washington University in St. Louis (WASH U-HCP3T).

**Supl. Table 3. Literature-based NTS connectivity**

| **Medulla oblongata** | | **Cortex** | | | **Thalamus/Subthalamus** | |
| --- | --- | --- | --- | --- | --- | --- |
| ambiguus nucl. | 2,3,4,6,8 | insula | 2,5,6,7,8 | | unspecific-thalamus | 2,3,4,7,8 |
| dorsal motor nucl. n. X | 2,4,5,6,8 | operculum | 2,8 | | paraventricular thalamic nucl. | 2,7 |
| RVLM | 3,4,5,6 | *-pars opercularis, inf. frontal gyrus* | 2,4 | | ventral posteromedial nucl. | 2 |
| CVLM: | 3,5,6 | **Frontal lobe:** |  | | dorsomedial nucl. | 2 |
| *-caudal pressor area* | 5 | medial prefrontal cortex | 2,4,5,7,8 | | pulvinar nucl. | 2 |
| *-caudal midline medulla* | 3 | dorsolateral prefrontal cortex | 5,8 | | zona incerta | 2 |
| nucl. spinalis n. V | 1,2,3,6,8 | frontal orbital cortex | 2,8 | | **Hippocampus** | |
| paratrigeminal nucl. | 1,2 | ventral orbital cortex | 2 | | unspecific-hippocampus | 2,4,7,8 |
| hypoglossus nucl. | 6,8 | lateral orbital cortex | 2 | | dorsal/ventral subiculum | 2,7 |
| inf. olivary nucl. | 3,6 | infralimbic cortex | 2 | | parahippocampus | 8 |
| dorsoventral olivary nucl. | 8 | prelimbic cortex | 2 | | **Hypothalamus** | |
| gracilis nucl. | 8 | superior frontal gyrus | 8 | | unspecific-hypothalamus | 1,2,3,4,6,7,8 |
| cuneate nucl. | 8 | supramarginal gyrus | 8 | | -dorsomedial hypothalamus | 2,3,5 |
| preBötzinger complex | 8 | middle frontal gyrus | 8 | | -ventromedial hypothalamus | 2,3 |
| parapyramidal region | 3 | precentral gyrus | 8 | | -lat. hypothalamus | 2 |
| area postrema | 3 | prim. motor cortex | 2,5 | | -arcuate nucl. | 2 |
| medullary raphe | 5 | supplementary motor area | 2,8 | | -posterior hypothalmus | 2 |
| raphe pallidus nucl. | 2 | sec. motor cortex | 2 | | -medial preoptic area | 2 |
| raphe magnus nucl. | 2 | premotor cortex | 2 | | -anterior hypothalamus | 3 |
| raphe obscurus nucl. | 6 | **Cingulate cortex:** |  | | **Amygdala** | |
| -lat. reticular nucl. | 2,6 | anterior cingulate cortex (ACC) | 2,5,7,8 | | unspecific-amygdala | 2,3,5,6,7,8 |
| -lat. paragigantocellular nucl. | 2,6 | *-perigenual ACC* | 5 | | stria terminalis | 2,8 |
| -gigantocellular ret. nucl. | 2 | mid-cingulate cortex | 2,5,8 | | **Basal ganglia** | |
| -central reticular nucl. | 4 | posterior cingulate cortex | 8 | | accumbens nucl. | 2,7,8 |
| -intermediate reticular nucl. | 6 | paracingulate | 8 | | putamen | 2,8 |
| -dors. paragigantocellular nucl. | 6 | retrosplenial cortex | 4 | | caudate | 2,8 |
| **Pons** | | **Parietal lobe:** | |  | globus pallidus | 2,8 |
| parabrachial nucl. | 2,4,7,8 | prim. somatosensory cortex | 2,4,5,8 | | substantia nigra | 2,8 |
| *-lat. parabrachial nucl.* | 2 | sec. somatosensory cortex | 5,8 | | corpus callosum | 8 |
| locus coeruleus | 2,4,8 | precuneus | 5,8 | | fornix | 8 |
| pontine nucl. | 4,8 | postcentral gyrus | 2,8 | | ventral pallidum | 2 |
| dorsolateral pons | 3,6 | angular gyrus | 8 | | internal capsule | 3 |
| vestibular area | 3 | **Rhinenzephalon:** |  | |  |  |
| spinal vestibular nucl. | 6 | piriform cortex | 2 | |  |  |
| vestibular nucl. | 8 | **Temporal lobe:** |  | |  |  |
| parvocellular reticular nucl. | 2 | transverse temporal gyri | 8 | |  |  |
| pontine reticular nucl. | 2 | superior temporal gyrus | 8 | |  |  |
| **Mesencephalon** | | middle temporal gyrus | | 8 |  |  |
| periaqueductal gray | 3,5,8 | entorhinal area | 8 | |  |  |
| tegmental nucl. | 2 | lateral occipito-temporal gyrus | 8 | |  |  |
| *-ventral tegmental area* | 2,8 | medial occipito-temporal gyrus | 8 | |  |  |
| ant. pretectal area | 3 | **Occipital lobe:** |  | |  |  |
| sup. colliculus | 3 | lateral occipital cortex | 8 | |  |  |
| reticular area midbrain | 3 | inferior occipital cortex | 8 | |  |  |
| red nucleus | 8 | middle occipital cortex | 8 | |  |  |
| med. longitudinal fasciculus | 8 | superior occipital cortex | 8 | |  |  |
| cuneiformis nucl. | 8 | **Cerebellum** | | |  |  |
| raphe dorsal nucl. | 8 | cerebellar hemisphere | 2,4,5,8 | |  |  |
| raphe median nucl. | 8 | cerebellar vermis | 2,3,6 | |  |  |
| **Dienzephalon** | | 2^nd^ cerebellar lobule | | 3 |  |  |
| habenula | 2 | 3^rd^ cerebellar lobule | 3 | |  |  |
| mamillary body | 2 | 4^th^ cerebellar lobule | 3 | |  |  |
|  |  | 5^th^ cerebellar lobule | 3 | |  |  |
|  |  | fastigial nucl. | 3 | |  |  |
|  |  | interpositus nucl. | 3 | |  |  |
|  |  | dentate nucl. | 3 | |  |  |
|  |  | superior cerebellar peduncle | 8 | |  |  |

Indicated is the literature based extraction of ROIs showing functional connectivity to the NTS. The regions in grey show functional or structural involvement of the NTS within at least three out of eight different functional organ systems. The identification of regions was based on the literature selected via the systematic review (see Supl. Table 1).

**Organ Systems Key:** 1 = RTS = Respiratory/Tracheolaryngeal System; 2 = GGS = Gustatory/Gastroesophageal System; 3 = MHB = Metabolism/Hormonal Balance; 4 = CVM = Cardiovagal Modulation; 5 = MSNA = Muscle Sympathetic Nerve Activity; 6 = BARO= Baroreflex; 7 = SCI = Spinal cord injury; 8 = AVM = Auriculovagal Modulation

**Supl. File. 1. Axial NTS slices.**


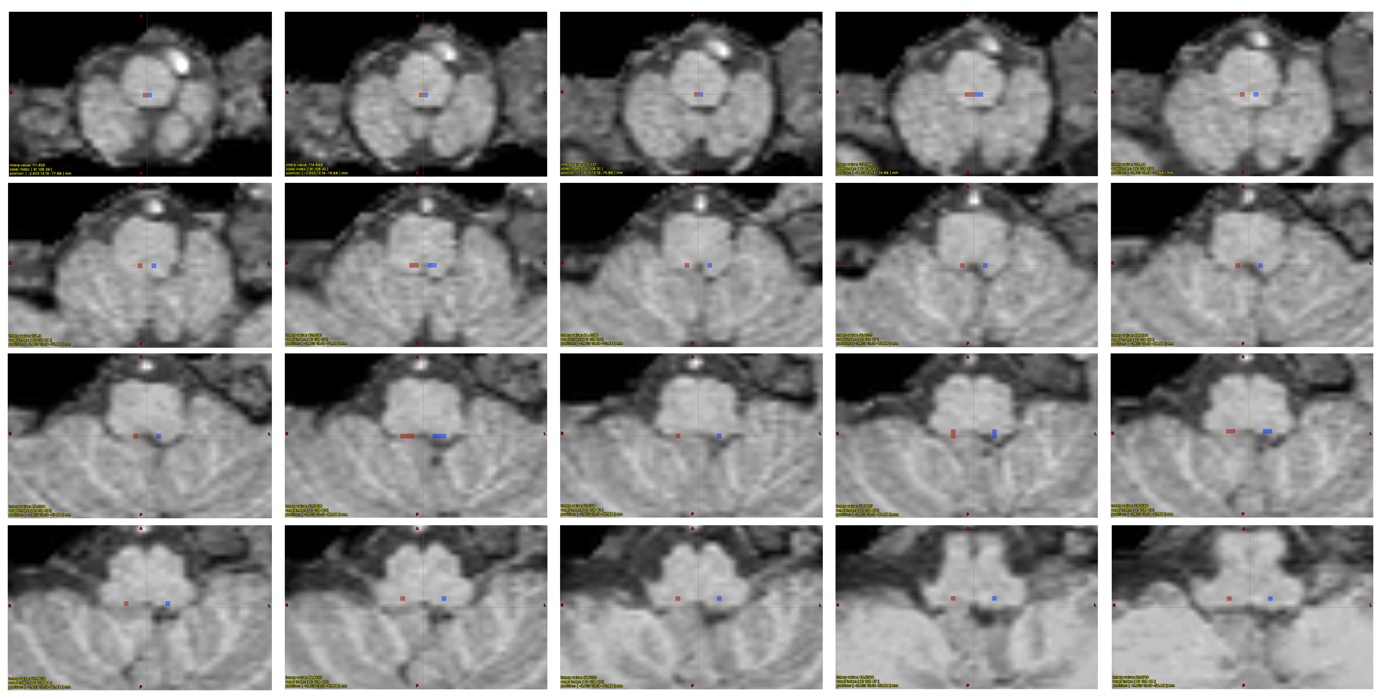


Displayed are the axial slices of one representative individual (subj. ID 101107 in the HCP database). The entire length of the right (red) and the left (blue) NTS starting from the most caudal part (left upper corner) towards the most rostral part of the NTS (right lower corner) is displayed.

**Supl. File. 2. 3D models key**

Displayed is the color coded key of brainstem, subcortical and cortical regions.

Brainstem regions:


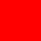
 NTS


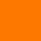
 NA


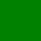
 CVLM


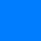
 RVLM


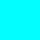
 LC


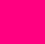
 SpV


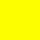
 Xmn


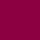
 PBC


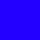
 PAG

Subcortical regions:


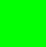
 THAL_AG


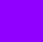
 THAL_IG


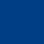
 THAL_LG


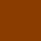
 THAL_MG


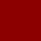
 THAL_PG


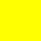
 THAL_VG


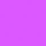
 AMY_AAA


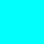
 AMY_ABN


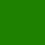
 AMY_BN


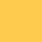
 AMY_CEN


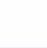
 AMY_CON


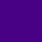
 AMY_COR


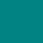
 AMY_LN


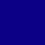
 AMY_MN


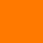
 AMY_PLN


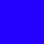
 NAC


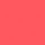
 HIPPO


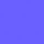
 HYP_ant_sup


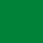
 HYP_ant_inf


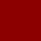
 HYP_int


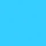
 HYP_post

Cortical regions:


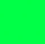
 ACC


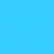
 INS


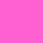
 MCC


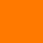
 mPFC


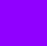
 S1

**Supl. File. 3. 3D model subcortical-brainstem**

Displayed are the brainstem and subcortical regions within a 360° horizontal view.

**Supl. File. 4. 3D model cortical-brainstem**

Displayed are the brainstem and cortical regions within a 360° horizontal view.
